# Supplementary material for: An improved pig reference genome sequence to enable pig genetics and genomics research
Source: Gigascience. 2020 Jun 16;9(6):giaa051. doi: 10.1093/gigascience/giaa051 (PMC7448572; doi:10.1093/gigascience/giaa051)

Supplementary methods, tables and figures

1. Sequence and assembly....3

1.1 From Duroc 2-14 DNA to Sscrofa11.1 assembly....3

1.1.1 Sample, sequencing and assembly....3

1.1.2 Contig quality assessment and contig splitting....3

1.1.3 Scaffolding....3

1.1.4 Gap filling....3

1.1.5 Targeted BAC sequencing to fill gaps....4

1.1.6 Polishing....4

1.2 From MARC1423004 DNA to assembly USMARCv1.0....4

1.2.1 Sample, sequencing and assembly....4

1.2.2 Scaffolding....6

1.2.3 Gap filling....6

1.2.4 Polishing....6

1.3 Anchoring the assemblies to chromosomes....6

1.3.1 Chromosome Preparation....6

1.3.2 Preparation and Selection of BAC clones for FISH....6

1.3.3 Fluorescence *in situ* hybridisation....6

2. Annotation (Ensembl)....7

2.1 Repeat Finding....7

2.2 Low complexity features, ab initio predictions and BLAST analyses....7

2.3 Protein-coding model generation....7

2.3.1 cDNA alignment....7

2.3.2 Projection mapping pipeline....7

2.3.3 Iso-Seq aligment....7

2.3.4 Protein-to-genome alignment....8

2.3.5 RNA-seq alignment....8

2.3.6 Immunoglobulin and T-cell receptor genes....8

2.3.7 Selenocysteine proteins....8

2.3.8 Filtering the models....8

2.3.9 Collapsing the transcript set....9

2.3.10 Addition of UTR to coding models....9

2.3.11 Generating multi-transcript genes....9

2.3.12 Pseudogenes....9

2.3.13 Small ncRNAs....10

2.3.14 lncRNAs identification....10

2.3.15 Cross-referencing and stable identifiers ... 10
2.3.16 Gene expression ... 10
2.3.17 Comparison of Ensembl and NCBI annotation ... 10
2.4 Annotation of non-reference pig assemblies ... 10
2.4.1 Annotation of 11 non-reference pig breed assemblies ... 10
2.4.2 Annotation of the USMARCv1.0 assembly ... 10
3. References ... 11
Supplementary Tables and Figures ... 12

1. Sequence and assembly

1.1 From Duroc 2-14 DNA to Sscrofa11.1 assembly

1.1.1 Sample, sequencing and assembly

DNA was extracted from Duroc 2-14 cultured fibroblast cells passage 16-18 using the Qiagen Blood & Cell Culture DNA Maxi Kit, producing 139.15 µg DNA from three extractions. The high molecular weight DNA from this extraction was sequenced by Pacific Biosciences (PacBio) using their long read sequencing technology. Libraries for SMRT sequencing were prepared and sequenced as described previously [60] using P6-C4 chemistry on the RSII using 213 SMRT cells.

Contigs were assembled using the Falcon v0.4.0 assembly pipeline following the standard protocol. Quiver v. 2.3.0 [61] was used to correct the primary and alternative contigs. Only the primary pseudo-haplotype contigs were used in the assembly.

1.1.2 Contig quality assessment and contig splitting

Paired-end Illumina reads from the same individual (http://www.ebi.ac.uk/ena/data/view/PRJEB9115) were mapped to the 3,206 haploid contigs and assessed for structural abnormalities using the methods described previously [14]. Briefly, 1,000 bp windows across the contigs were assessed for levels of abnormal mapping including high GC-normalized coverage, improper pairing and unexpected insert sizes. Additionally BAC end sequences (BES) (CHORI-242 library) [12] and fosmids (WTSI_1005 library: https://www.ncbi.nlm.nih.gov/clone/library/genomic/234/) (ENA accession: HE000001 – HE565349) [20] from the same individual (i.e. Duroc 2-14) were mapped to the contigs and regions with multiple occurrences of incorrect orientation were examined manually in the Integrative Genomics Viewer (IGV) [80]. For 28 contigs where there was consistent evidence of structural disagreement between the contigs and the Illumina reads, BAC ends and fosmids, the contigs were split or trimmed.

1.1.3 Scaffolding

In order to establish an initial scaffold the contigs were mapped to Sscrofa10.2 using Nucmer (v3.23) [28]. The positioning of the contigs was determined by using the longest ascending subset of mapping locations using the show-coords tool from Mummer with the –g flag. Contigs with a %IDY below 95% were excluded. Contigs that mapped to regions substantially larger (>180%) or smaller (<10%) than the contig size were excluded. These tolerances were intentionally lenient due to the inflated gap sizes in the Sscrofa10.2 assembly (e.g. including 50 kb between scaffolds as required by the NCBI submission system in 2011) and highly fragmented nature of certain regions of Sscrofa10.2. Adjacent contigs were merged into a single fasta entry with Ns representing gaps between them. Gaps were estimated from the distance between the mapping locations against Sscrofa10.2, with an upper limit of 50 kb. Several of the remaining contigs were placed by identifying their longest alignment position, if this alignment was more than 50% the length of the contig and overlapped with a gap with a IDY>90% they were placed in the gaps with 25 bp gaps either side. 346 contigs covering 2.3 Gb were included in the initial chromosomal scaffolds.

1.1.4 Gap filling

PBJelly [29] was used with the 65X raw PacBio reads to fill the gaps in the scaffolds. Default parameters were used for all stages except the assembly stage where max wiggle (-w) was set to 100 kb and max trim (-t) was set to 1,000 bp. These parameters were changed to account for the extremely inaccurate gap sizes and missing sequence in Sscrofa10.2 that will have influenced the estimated gap sizes, to allow heavily overlapping contigs to be closed and to allow potentially low-quality sequence at the end of contigs to be excluded. Following initial gap filling, PBJelly was rerun on the fasta output from the first round, with the unused contigs from the Falcon output added to the fasta to allow extension of the scaffolds. These contigs had been excluded initially to reduce secondary mapping positions. PBJelly is able to add

contigs to the end of scaffolds, but not place whole contigs in gaps, so the initial mapping of contigs to scaffolds was examined to find if any of the contigs that had been excluded in this stage due to overlap with existing contigs might fill the gaps. Contigs were placed on a case-by-case basis if there was evidence of overlap with placed sequence on both sides of the gap, if the initial contig quality control was good, and if placement was well supported by BAC end mapping. Additionally, BACs for which the end sequences mapped to adjacent contigs providing evidence for scaffolding these adjacent contigs and for which finished quality sequence was publically available, were aligned and the gap filled and placed following the same restrictions as the unplaced contigs. On completion of these gap-filling procedures 108 gaps remained. Estimation of the size of the remaining gaps was based on BAC end mapping, using the known median insert size of the CHORI-242 library [25]. Any gaps estimated to be <100 bp were sized at 100 bp and unspanned gaps were sized at 50 kb.

1.1.5 Targeted BAC sequencing to fill gaps

Five BACs from the CHORI-242 library were selected for further sequencing (CH242-188M9 (SSC16); CH242-323K10 (SSC18); CH242-284F8 (SSC18); CH242-61K12 (SSC1); CH242-168C15 (SSC12)) based on BAC ends mapping either side of gaps [12]. The BAC clones were obtained from BACPAC [25] and DNA was extracted using the Epicentre BACMAX DNA purification kit following manufacturer's instructions. The BAC DNA was sequenced using Oxford Nanopore Technologies' MinION sequencer using a barcoded 2D library following the discontinued protocol SQK-LSK208 on an R9 flow cell using MinKNOW v1.0.5. Sequences were assembled using Canu [81] with default settings and each produced a single contig. The BAC vector sequences were removed from the contigs, the contigs were mapped to the assembly initially with Nucmer to confirm they mapped to the expected locations, with exact positions for placement determined by BWA-MEM [82]. All five contigs mapped to the expected positions and were placed to close the targeted gaps, leaving 103 gaps in the final Sscrofa11 assembly and closing chromosomes 16 and 18.

1.1.6 Polishing

Error correction was done using Arrow from the GenomicConsensus suite (https://github.com/PacificBiosciences/GenomicConsensus) using the original 65X PacBio coverage. This was followed by Pilon [83] with fixlist restricted to "bases", but otherwise using default parameters and paired-end Illumina short read data that provided 50x genome coverage.

1.2 From MARC1423004 DNA to assembly USMARCv1.0

1.2.1 Sample, sequencing and assembly

DNA was isolated from barrow MARC1423004 using a salt extraction method. Briefly, frozen lung tissue was crushed into powder, scraped into a 15 mL tube, and suspended in 4 mL digestion buffer (10 mM NH_4Cl, 400 mM NaCl, 50 mM Na_2EDTA, pH 8.0). Digestion was initiated with 100 µL 20% SDS and 70 µL trypsin (5 mg/ml). This initial digestion was allowed to proceed at room temperature (approximately 22ºC) for one hour, and then 200 µL of 20% SDS and 50 µL of Proteinase K (50 mg/mL) were added. The digestion was incubated at 55ºC in a shaking water bath overnight (16 hours). Another 100 µL of Proteinase K were added and incubation extended for another 1.5 hours, until no remaining tissue pieces could be observed in the solution, and then 10 µL of RNase (10 U/µL) were added followed by additional incubation for one hour. 1.25 mL 5M NaCl was added, mixed by inversion, and the tube was centrifuged at 3200 x g at 4ºC. The supernatant was transferred to a fresh 15 mL tube, and DNA precipitated by addition of 2.5 volumes of 95% ethanol. The precipitate was removed using a hooked Pasteur pipet, dipped twice in separate tubes of 70% ethanol on ice, and allowed to briefly dry in air on the hook. The DNA was then eluted from the hook by placing it under 250 µL TE buffer (10 mM Tris-HCl, 0.1 mM EDTA) until the pellet slipped off into the buffer. The hook was then removed, and the DNA was allowed to dissolve into the buffer for several days at 4ºC until it appeared to be completely dissolved. The high molecular weight

DNA from this extraction was sequenced by Pacific Biosciences (PacBio) using their long read sequencing technology. Libraries for SMRT sequencing were prepared and sequenced as described previously [60] using P5/C3 and P6-C4 chemistry on the RSII. A total of 199 P5/C3 cells and 127 P6/C4 cells were produced. Initial read statistics are detailed in Table S1. Contigs were assembled using Celera Assembler v8.3rc2 [32] using the command:

```
wgs-8.3/Linux-amd64/bin/PBcR -s pacbio.spec -fastq
filtered_subreads.fastq genomeSize=3000000000 -sensitive -l swine
sgeName=swine "sge=-p -500 -A swinenewsens"  useGrid=1 scriptOnGrid=1

and spec file:
merSize = 16

ovlMemory = 32
ovlStoreMemory = 32000
ovlThreads = 32
threads = 32
ovlConcurrency = 1
cnsConcurrency = 8
merylThreads = 32
merylMemory = 32000
frgCorrThreads = 16
frgCorrBatchSize = 100000
ovlCorrBatchSize = 100000

useGrid=1
scriptOnGrid=1
ovlCorrOnGrid=1
frgCorrOnGrid=1

sge = -A assembly
sgeScript = -pe threads 1
sgeConsensus = -pe threads 8
sgeOverlap = -pe threads 4 -l mem=2GB
gridEngineMhap = -pe threads 15 -l mem=2GB
sgeCorrection = -pe threads 15 -l mem=2GB
sgeOverlapCorrection  = -pe threads 1 -l mem=16GB
sgeFragmentCorrection=-pe threads 2 -l mem=2GB
sgeOverlapCorrection=-pe threads 1 -l mem=4GB

asmOvlErrorRate=0.1
asmUtgErrorRate=0.06
asmCgwErrorRate=0.1
asmCnsErrorRate=0.1
asmOBT=1
asmObtErrorRate=0.08
asmObtErrorLimit=4.5

batOptions=-RS -NS -CS
utgGraphErrorRate=0.055
utgGraphErrorLimit=4
utgMergeErrorRate=0.055
utgGraphErrorLimit=4

ovlHashBits=24
ovlHashLoad=0.80

ovlHashBlockLength      =300000000
ovlRefBlockLength       =0
ovlRefBlockSize         =2000000
```

This initial assembly was 2.67 Gbp in 16,441 contigs and an N50 of 2.8 Mbp. Quiver from SMRTportal v. 2.3.0 [61] was used to correct the assembly.

1.2.2 Scaffolding

The lung tissue from the pig was sent to Dovetail Genomics (Santa Cruz) for scaffolding by Chicago and HiRise as described [26]. This process identified 270 putative misjoins in the contigs and output scaffolds 13,039 scaffolds (294 > 50 kb). The total length was 2.66 Gbp and scaffold N50 was 36.5 Mbp. The dovetail scaffolds were gap-filled where a single contig spanned the gap, correcting false breaks made by HiRise. The resulting assembly was used for reference-guided scaffolding based on the Sscrofa11.1 reference. In case of conflicts, with the exception of cross-chromosome joins, the USDA assembly was unchanged.

1.2.3 Gap filling

PBJelly [29] was used with the 65X raw PacBio reads to fill the gaps in the scaffolds. Default parameters were used for all steps.

1.2.4 Polishing

Gap filling was followed by Pilon [83] with fixlist restricted to "bases", but otherwise using default parameters and paired-end Illumina short read data that provided 50x genome coverage. The final assembly of 2.8 Gbp has a scaffold N50 of 131.5 Mbp and a contig N50 of 6.4 Mbp (Table 1).

1.3 Anchoring the assemblies to chromosomes

1.3.1 Chromosome Preparation

Heparinized blood samples were cultured for 72 h in PB MAX Karyotyping medium (Invitrogen) at 37°C, 5% CO_2. Cell division was arrested by adding colcemid at a concentration of 10.0 µg/ml (Gibco) for 30 min prior to hypotonic treatment with 75 mM KCl and fixation to glass slides using 3:1 methanol:acetic acid.

1.3.2 Preparation and Selection of BAC clones for FISH

BAC clones with inserts of approximately 150 kb in size were selected for position using the Sscrofa10.2 NCBI database (www.ncbi.nim.nih.gov) and ordered from the PigE-BAC library (ARK-Genomics) [84] and the CHORI-242 Porcine BAC library [25]. BAC clone DNA was isolated using the Qiagen Miniprep Kit (Qiagen) prior to amplification and direct labelling by nick translation. Probes were labelled with Texas Red-12-dUTP (Invitrogen) and FITC-Fluorescein-12-UTP (Roche) prior to purification using the Qiagen Nucleotide Removal Kit (Qiagen).

1.3.3 Fluorescence *in situ* hybridisation

Metaphase preparations were fixed to slides and dehydrated through an ethanol series (2 mins each in 2×SSC, 70%, 85% and 100% ethanol at RT). Probes were diluted in a formamide buffer (Cytocell) with Porcine Hybloc (Insight Biotech) and applied to the metaphase preparations on a 37°C hotplate before sealing with rubber cement. Probe and target DNA were simultaneously denatured for 2 mins on a 75°C hotplate prior to hybridisation in a humidified chamber at 37°C for 16 h. Slides were washed post hybridisation in 0.4x SSC at 72°C for 2 mins followed by 2x SSC/0.05% Tween 20 at RT for 30 secs, and then counterstained using VECTASHIELD anti-fade medium with DAPI (Vector Labs). Images were captured using an Olympus BX61 epifluorescence microscope with cooled CCD camera and SmartCapture (Digital Scientific UK) system.

2. Annotation (Ensembl)

2.1 Repeat Finding

After loading into a database, the pig genome assemblies, including the Sscrofa11.1 reference genome sequence were screened for sequence patterns, including repeats using RepeatMasker [62] (version 4.0.5) with parameters '-nolow –species "sus_scrofa" –engine "crossmatch"', Dust (Kuzio, J., R. Tatusov, and D. Lipman, *Dust.* Unpublished but briefly described in [85]) and TRF [65]. For the pig genome annotation, the Repbase pig library was used with Repeatmasker.

2.2 Low complexity features, ab initio predictions and BLAST analyses

Transcription start sites (TSS) were predicted using Eponine-scan [86]. CpG islands [Micklem, G., unpublished] longer than 400 bases and tRNAs [87] were also predicted. The results of Eponine-scan, CpG and tRNAscan are for display purposes only and are not used subsequently in the gene annotation process. Genscan [88] was run across the repeat-masked sequence to identify *ab initio* gene predictions. The Genscan predictions were used as a supplementary data track, but not used further in the main annotation.

2.3 Protein-coding model generation

Various sources of transcript and protein data were investigated and used to generate gene models using a variety of techniques and are outlined below. Potential transcript models from each evidence source were clustered and each locus was analysed to select the most likely set of full length isoforms, with preference given to species-specific data over data based on homology (described in more detail in section 2.3.9).

2.3.1 cDNA alignment

cDNAs were downloaded from ENA (www.ebi.ac.uk/ena) and RefSeq [89], and aligned to the genome using Exonerate [90]. Only known mRNAs were used (NMs). A minimal sequence length of 60 bp was used and a cut-off of 97% identity and 90% coverage were required for an alignment to be processed further. Open reading frame potential was assessed via a BLAST of all UniProt protein existence level 1 and 2 vertebrate proteins.

2.3.2 Projection mapping pipeline

Whole genome alignments against the human GRCh38.p12 genome assembly were generated using LastZ. Regions of conserved synteny identified using these alignments were used to map protein-coding annotation from the Ensembl/GENCODE [91] gene set. For each protein-coding gene in the human genome, we projected the coding exons within the canonical transcript to pig. In case of exonic overlap on the projected sequence, the longest exon took precedence. Where the mapping did not succeed, we selected the next successful projection of the transcript having the longest translation.

2.3.3 Iso-Seq aligment

PacBio Iso-Seq data are high coverage long read transcriptomic data that allows for correction for the high error rate in raw PacBio reads. The consensus sequences representing nine tissues (brain, diaphragm, hypothalamus, liver, skeletal muscle (*longissimus dorsi*), pituitary, small intestine, spleen, and thymus were downloaded from the short read archive (SRA: PRJNA351265) after correction using Illumina short reads from the same tissue type. The sequences were aligned to the genome using Minimap2 [92] with the -splice:hq parameter. Mappings were subsequently filtered to remove sequences with < 90 percent identity and < 95 percent coverage to the reference sequence. All the Iso-Seq data sets had 3' capping and were used for adding UTRs to homology-based protein coding models. All Iso-Seq data sets were used as lncRNA candidates for our lncRNA prediction pipeline.

2.3.4 Protein-to-genome alignment

Protein sequences were downloaded from UniProt and aligned to the genome in a splice aware manner using GenBlast [93]. The set of proteins aligned to the genome was a subset of UniProt proteins used to provide a broad targeted coverage of the pig genome. The set consisted of the following:

- Pig SwissProt/TrEMBL PE level 1, 2, 3
- Human SwissProt/TrEMBL PE level 1, 2
- Mouse SwissProt/TrEMBL PE level 1, 2
- Other mammals SwissProt/TrEMBL PE level 1, 2

Note: PE level = protein existence level. A cut-off of 50 percent coverage and identity and an e-value of e-1 were used for GenBlast [93] with the exon repair option turned on. The top 5 transcript models built by GenBlast for each protein passing the cut-offs were kept.

2.3.5 RNA-seq alignment

RNA-Seq data were downloaded from ENA (https://www.ebi.ac.uk/ena/) and used in the annotation. RNA-Seq data from PRJEB19386 and PRJEB33353 were used for annotation. The PRJEB19386 RNA-Seq data consisted of 150 bp paired end reads from libraries prepared using a stranded library protocol from ribo-depleted total RNA from Duroc pigs. The PRJEB19386 dataset comprised RNA-Seq data from 28 tissue and cell samples: alveolar macrophages, amygdala, brain stem, caecum, cerebellum, colon, corpus callosum, duodenum, epididymis, frontal lobe (brain), hippocampus, ileum, kidney cortex, left ventricle (heart), mesenteric lymph node, medulla oblongata, occipital lobe, omentum, penis, pituitary gland, pons, skeletal muscle, spleen, stomach, thalamus, tonsil, uterus (Table S10). The PRJEB33353 data set consisted of four tissues brain, muscle, liver and testes. A merged file containing reads from all tissues was also created. The merged data were less likely to suffer from model fragmentation due to read depth. The available reads were aligned to the genome using BWA. A 50 percent allowed mismatch criteria was applied to identify potential splice junctions. Initial rough exon/intron boundaries were generated via the BWA alignments and then refined by mapping the reads in a splice-aware manner using Exonerate [90]. Protein-coding models were identified from BLAST alignments of the longest ORF against the UniProt vertebrate PE1 & 2 dataset. Models with poorly scoring or no BLAST alignments were split into a separate class and considered as potential lncRNAs.

2.3.6 Immunoglobulin and T-cell receptor genes

Translations of different human IG gene segments were downloaded from the IMGT database [94] and aligned to the genome using GenBlast [93]. For the pig annotation, logical thresholds for coverage (80%), percent identity (70%) and e-value (E-1) were used for GenBlast with the exon repair option on. The top 10 transcript models built by GenBlast for each protein passing the cut-offs were kept.

2.3.7 Selenocysteine proteins

Known selenocysteine proteins were aligned against the genome using Exonerate [90]. The models generated were checked for the presence of selenocysteines in the same positions as the known proteins. We only kept models with at least 90% coverage and 95% identity.

2.3.8 Filtering the models

The filtering phase decided the subset of protein-coding transcript models, generated from the model-building pipelines, that would comprise the final protein-coding gene set in the GeneBuild. Models were filtered based on information such as what pipeline they were generated using, how closely related the data are to the target species (i.e. pig) and how good the alignment coverage and percent identity to the original data are. Models were filtered using the LayerAnnotation and GeneBuilder modules. The Apollo software [95] was used to visualise the results of the filtering.

2.3.9 Collapsing the transcript set

The LayerAnnotation module was used to define a hierarchy of input data sets, from most preferred to least preferred. The output of this pipeline included all transcript models form the highest ranked input set. Models from lower ranked input sets were included only if their exons do not overlap a model from an input set higher in the hierarchy. Note that models cannot exist in more than one layer. For UniProt proteins, models were also separated into clades, to help selection during the layering process.

Each locus was examined in terms of total evidence. The highest priority was given to specialised gene types, the IG/TR genes and selenocysteine genes. After this, priority was given to unique transcript isoforms derived from transcriptomic data (RNA-seq and Iso-Seq) and cDNA sequences that had an open reading frame with a high level of coverage (90 percent or more) to a PE12 vertebrate protein. Gap filling was carried out using homology data from both the human annotation mappings and the UniProt alignments (again with preference for models with 90 percent coverage when re-aligned to the original evidence). After this, potential fragments were added in, again with preference given to the transcriptomic data and cDNAs over the homology data.

A detailed breakdown of the layers used can be found here:
https://github.com/Ensembl/ensembl-analysis/blob/feature/pig_98_layers/modules/Bio/EnsEMBL/Analysis/Hive/Config/LayerAnnotationStatic.pm

2.3.10 Addition of UTR to coding models

Models built from homology data lacked untranslated regions (UTRs). In order to call potential UTRs for these cases we used the available cDNA, long and short read data. When multiple UTR donors were available at a locus, the source of the UTR was prioritised with UTR coming from cDNAs and Iso-Seq, then RNA-seq, with a minimum requirement the terminal intron of any spliced models lacking UTR needed to be identical to an intron in the potential UTR donor. If multiple alternative UTRs were available for the highest priority source, suitability of a UTR match was based on the splicing pattern of the UTR donor versus the splicing pattern of the target mode. The donor with the highest number identical splice junctions to the target was selected. If the acceptor ORF was originally missing a start or a stop codon, the ORF was examined for possible extension via the added UTR sequence (while maintaining the frame of the ORF). Single exon ORFs had UTR addition based on residing fully within a potential UTR donor sequence.

2.3.11 Generating multi-transcript genes

The steps described above generated a large set of potential transcript models, many of which overlapped one another. Redundant transcript models were collapsed and the remaining unique set of transcript models were clustered into multi-transcript genes where each transcript in a gene has at least one coding exon that overlaps a coding exon from another transcript within the same genes.

2.3.12 Pseudogenes

Pseudogenes were annotated by looking for genes with evidence of frame-shifting or genes located in regions rich in repetitive sequences. Introns of less than 75 bp length were flagged as potential artificial introns. Transcripts with 2 or more suspect introns or transcripts where >= 80 percent of the introns were covered by repeats where flagged as evidence of a potential pseudogene. Single exon retrotransposed pseudogenes were identified by searching for a multi-exon equivalent elsewhere in the genome.

2.3.13 Small ncRNAs

Small non-coding (sncRNA) genes were added using annotations taken from RFAM [96] and miRBase [97]. BLAST was run for these sequences to identify homologs in the genome sequence and models were evaluated for expected stem-loop structures using RNAfold [98]. Additional machine learning based filters were applied to exclude predictions with sub-optimal alignments to the genome and non-conforming secondary structures. For other sncRNAs, models were built using the Infernal software suite [99].

2.3.14 lncRNAs identification

Using the transcriptomic data set, we tried to predict long intergenic non-coding RNAs (lncRNAs). We used the RNA-seq and Iso-Seq data which were filtered against the protein-coding gene set. Firstly, candidates with any overlap to a protein alignment evidence used to build the protein coding set were removed. Next sequences that were less than 200bp were removed. Due to the amount of transcriptional noise present in the Iso-Seq a final level of filtration was carried out. Here candidates with less than two splice junctions were removed, unless they had covered at least 1000bp of aligned sequence.

2.3.15 Cross-referencing and stable identifiers

Before public release the transcripts and translations were given external references (cross-references to external databases). Stable identifiers were assigned to each gene, transcript, exon and translation. As earlier pig genome sequences have been annotated by Ensembl previously a comparison was made to the previous gene set and as many stable identifiers as possible were mapped between the two annotations.

2.3.16 Gene expression

The Illumina RNA-Seq data (Table S10) were also processed by the EBI Gene Expression Atlas (GXA) team [100] (https://www.ebi.ac.uk/gxa/home) to generate a baseline gene expression atlas (Expression Atlas release 25, August 2017). These gene expression data can be visualised in the Ensembl genome browser from the gene page.

2.3.17 Comparison of Ensembl and NCBI annotation

The Sscrofa11.1 assembly was also annotated independently by the NCBI [43]. We have compared these two annotations (Table S8).

2.4 Annotation of non-reference pig assemblies

2.4.1 Annotation of 11 non-reference pig breed assemblies

Annotation for 11 other non-reference pig breed assemblies was also carried out using the Ensembl pipeline [38, 39]. The breeds included: Bamei, Berkshire, Hampshire, Jinhua, Landrace, Large White, Meishan, Pietrain, Rongchang, Tibetan and Wuzhishan (minipig) (Table S6). Annotation was carried out using the same code as the reference annotation, using the steps described in the above paragraphs. The only notable differences were: the ‘mammals’ library was used for repeatmasking and the four tissue PRJEB33353 RNA-seq data set was not used in the breed annotations. These differences are not expected to have had any noticeable effect on the overall result as the tissues in the PRJEB33353 data were already well represented in the other transcriptomic data used.

2.4.2 Annotation of the USMARCv1.0 assembly

Annotation for USMARCv1.0 was carried out using the Ensembl pipeline [38] and the same key steps as outlined for Sscrofa11.1. As the annotation of USMARCv1.0 was carried out prior to the latest reference annotation, the underlying code used had minor differences. These included the use of the ‘mammals’ library for repeatmasking, using Exonerate instead of Minimap2 for the Iso-Seq alignment and the four tissue PRJEB33353 RNA-seq data set was not used for the USMARCv1.0 annotation. These changes are not expected to have resulted in major differences in terms of the overall results.

3. References

Note: references 1-79 as cited in this document are listed in the main manuscript.

80. Robinson JT, Thorvaldsdóttir H, Winckler W, Guttman M, Lander ES, Getz G, et al. Integrative genomics viewer. Nat Biotechnol 2011;**29**:24–6.
81. Koren S, Walenz BP, Berlin K, JMiller JR, Bergman NH, Phillippy AM. Canu: Scalable and accurate long-read assembly via adaptive κ-mer weighting and repeat separation. Genome Res 2017;**27**:722–36.
82. Li H. Aligning sequence reads, clone sequences and assembly contigs with BWA-MEM. *ArXiv:1303.3997v1 [q-bio.GN]* (2013).
83. Walker BJ, Abeel T, Shea T, Priest M, Abouelliel A, Sakthikumar S, et al. Pilon: An integrated tool for comprehensive microbial variant detection and genome assembly improvement. PLoS ONE 2014;**9**:e112963.
84. Anderson SI, Lopez-Corrales NL, Gorick B, Archibald AL. A large-fragment porcine genomic library resource in a BAC vector. Mamm Genome 2000;**11**:811–4.
85. Morgulis A, Gertz EM, Schäffer AA, Agarwala R. A Fast and Symmetric DUST Implementation to Mask Low-Complexity DNA Sequences. J Comp Biol 2006;**13**:1028-40.
86. Down TA, Hubbard TJP. Computational detection and location of transcription start sites in mammalian genomic DNA. Genome Res 2002;**12**:458–61.
87. Lowe TM, Eddy SR. TRNAscan-SE: A program for improved detection of transfer RNA genes in genomic sequence. Nucleic Acids Res 1996;**25**:955–64.
88. Burge C, Karlin S. Prediction of complete gene structures in human genomic DNA. J Mol Biol 1997;**268**:78–94.
89. O'Leary NA, Wright MW, Brister JR, Ciufo S, Haddad D, McVeigh R, et al. Reference sequence (RefSeq) database at NCBI: current status, taxonomic expansion, and functional annotation. Nucleic Acids Res 2016;**44**:D733-45.
90. Slater GSC, Birney E. Automated generation of heuristics for biological sequence comparison. BMC Bioinformatics 2005;**6**:31.
91. Frankish A, Diekhans M, Ferreira AM, Johnson R, Jungreis I, Loveland J, et al. GENCODE reference annotation for the human and mouse genomes. Nucleic Acids Res 2019;**47**:D766-73.
92. Li H. Minimap2: pairwise alignment for nucleotide sequences. Bioinformatics 2018;**34**:3094-3100.
93. She R, Chu JS, Uyar B, Wang J, Wang K, Chen N. genBlastG: Using BLAST searches to build homologous gene models. Bioinformatics 2011;**27**:2141–3.
94. Lefranc MP, Giudicelli V, Duroux P, Jabado-Michaloud J, Folch G, Aouinti S, et al. IMGT®, the international ImMunoGeneTics information system® 25 years on. Nucleic Acids Res 2015;**43**:D413–22.
95. Lewis SE, Searle SMJ, Harris N, Gibson M, Iyer V, Richter J, et al. Apollo: a sequence annotation editor. Genome Biol 2002;**3**:research0082.1.
96. Griffiths-Jones S, Bateman A, Marshall M, Khanna A, Eddy SR. Rfam: An RNA family database, Nucleic Acids Res 2003;**31**:439–41.
97. Griffiths-Jones S, Grocock RJ, van Dongen S, Bateman A, Enright AJ. miRBase: microRNA sequences, targets and gene nomenclature. Nucleic Acids Res 2006;**34**:D140–4.
98. Lorenz R, Bernhart SH, Höner Zu Siederdissen C, Tafer H, Flamm C, Stadler PF, et al. ViennaRNA Package 2.0. Algorithms Mol Biol 2011;**6**:26.
99. Nawrocki EP, Eddy SR. Infernal 1.1: 100-fold faster RNA homology searches. Bioinformatics 2013;**29**:2933-5.
100. Papatheodorou I, Fonesca NA, Keays M, Tang YA, Barrera E, Bazant W, et al. Expression Atlas: Gene and protein expression across multiple studies and organisms. Nucleic Acids Res 2018;**46**:D246-51.

Supplementary Tables and Figures

Table S1. Pacific Biosciences read statistics

	TJTabasco (Duroc 2-14)	MARC1423004
**Chemistry**	P6/C4	P5/C3 and P6/C4
**Number of reads**	12,328,735	32,960,338
**Total length of reads (bp)**	175,934,815,397	186,973,885,772
**Mean read length (bp)**	14,270	6,144
**Read N50 (bp)**	19,786	9,277

Table S2. Predicted telomeres. Predicted telomere locations in the Sscrofa11.1 assembly. Number of exact matches of the vertebrate TTAGGG repeat sequence was used to identify candidate telomeres.

Chr	Start	End	Number of hexamers	Region length (kb)	Strand	Hexamer content
2	151,924,806	151,935,981	1,609	11.2	+	86.4%
3	132,840,959	132,848,913	1,046	8.0	+	78.9%
6	170,835,933	170,843,587	957	7.7	+	75.0%
8	138,963,948	138,966,197	208	2.2	+	55.5%
9	139,499,115	139,512,083	1,836	13.0	+	84.9%
14	141,745,369	141,755,446	1,201	10.1	+	71.5%
15	140,408,314	140,412,713	595	4.4	+	81.2%
18	55,971,782	55,982,971	1,571	11.2	+	84.2%
X	125,929,106	125,939,592	1,329	10.5	+	76.0%

Table S3. Predicted centromeres. Predicted centromere locations in the Sscrofa11.1 assembly.

Chr	Start	End	Repeat content (bp)	Region length (bp)	Repeat content
1	92,615,481	92,672,216	46,164	56,735	81.4%
1	92,760,768	92,881,119	110,990	120,351	92.2%
1	93,266,464	93,430,514	80,940	16,4050	49.3%
2	50,550,173	50,777,308	198,336	227,135	87.3%
3	41,776,737	41,860,603	35,376	83,866	42.2%
4	46,443,460	46,472,085	28,625	28,625	100.0%
5	39,774,025	39,828,563	54,538	54,538	100.0%
5	39,878,566	40,207,105	328,539	328,539	100.0%
6	38,712,705	38,886,534	163,335	173,829	94.0%
7	24,578,125	24,606,761	28,636	28,636	100.0%
8	144	20,905	20,761	20,761	100.0%
8	54,585,508	54,685,241	21,099	99,733	21.2%
9	63,144,551	63,503,859	356,770	359,308	99.3%
11	11,220,831	11,222,126	1,295	1,295	100.0%
11	35,726,738	35,728,355	1,617	1,617	100.0%
11	35,804,210	35,809,503	5,293	5,293	100.0%
11	35,870,705	35,878,206	7,501	7,501	100.0%
13	34	152,474	150,375	152,440	98.6%
15	1,649	36,105	10,369	34,456	30.1%
15	56,407,100	56,427,869	9,798	20,769	47.2%
17	63,189,675	63,361,433	171,758	171,758	100.0%
18	619	17,212	16,593	16,593	100.0%
Y	42,496,777	42,515,903	17,954	19,126	93.9%

Table S4. Assigning scaffolds to chromosomes. Fluorescent *in situ* hybridisation results using named BAC clones as probes plus sequence matches for sequences derived from these BAC clones.

Chr	BAC Name	BES	FISH	Sscrofa11.1 coordinates	USMARCv1.0 coordinates
1	PigE-232G23	CT070230.1; CT218278.1	1p	1:615,021-619,597	1:280,453,704-280,458,272
1	CH242-248F13	FP340244.3	1p	1:1,470,202-1,660,001	1:279,368,385-279,558,294
1	CH242-151E10	CT239299.1; CT245986.1	1q	unplaced scaffold: Contig1206	1: 6,156,768-6,336,737
2	PigE-117G14	CT074446.1; CT074447.1	2p	2:19,406-161,226	2:537,026-678,808
2	PigE-8G19	CT260033.1; CT260032.1	2p	2:552,031-671,098	2:29,620-146,529
2	CH242-188K23	CU929880	2 cen	2:52,747,463-52,933,130	2:51,728,649-51,908,148
2	CH242-230M23	CT144824.1; CT258059.1	2 cen	2:53,300,582-53,472,497	no match
2	CH242-441A1	CT364255.1;CT364256.1	2 cen	2:53,458,574-53,652,606	2:52,095,251-52,095,932
2	CH242-294F6	CT378635.1; CT378634.1	2q	2:151,178,736-151,402,963	2:145,456,152-145,678,427
3	PigE-168G22	CT094069.1; CT094070.1	3p	3:301,813-509,346	3:218,358-425,025
3	CH242-315N8	CT359002.1; CT359003.1	3q	3:122,720,374-122,869,530	no match
4	PigE-262E12	CT082779.1; CT193441.1	4p	4:37,383-223,717	4: 96,811- 97,511
4	PigE-131J18	CT116562.1; CT171811.1	4p	4:449,934-626,677	4:322,853-499,367
4	PigE-85G21	CT070098.1; CT190031.1	4q	4:130,625,653-130,748,215	4:130,215,908-130,338,404
5	CH242-288F8	CT132004.1; CT211915.1	5p	5:170,319-344,353	5:188,019-362,653
5	PigE-178M22	CT139068.1; CT155898.1	5p	5:175,168-311,462	5:192,886-329113
5	CH242-133F9	CT166002.1; CT166003.1	5p	5:438,296-633,458	5:456,924-652,458
5	PigE-127K14	CT057696.1; CT057697.1	5p	5:1,003,455-1,129,329	5:1,024,261-1,148,699
5	PigE-74P10	CT188857.1; CT188858.1	5p	5:3,739,938-3,883,755	5:103,338,585-103,481,984
5	PigE-99L23	CT079916.1; CT106700.1	5p Mid	5:31,980,969-32,114,628	no match
5	CH242-63B20	FP102738	5q	5:104,304,289-104,489,770	no match
6	PigE-238J17	CT220438.1; CT220439.1	6p	6:2,333,972-2,522,065	6:162,952,836-163,141,204
6	PigE-199E24	CT272854.1; CT272853.1	6 below cen	6:62,771,286-62,952,647	6:104,969,580-105,152,317
6	CH242-510F2	CT396711.1; CT442620.1	6q	6:170,248,061-170,454,571	6:162,654-369,119
7	PigE-52L22	CT054562.1; CT063652.1	7p	7:188,339-317,255	7:125,463,765-125,463,765
7	PigE-246A1	CT203984.1; CT070741.1	7 cen	7:24,628,314-24,671,828	no match
7	PigE-230H8	CT120917.1	7q below cen	7:46,704,415-46,704,995	7:395,704-396,284
7	PigE-75E21	CT188956.1; CT261917.1	7q below cen	7:46,901,592-47,032,091	7:68,406-199,212
7	CH242-103I13	CU695123.2	7q	Unplaced scaffold: Contig1914	7:7,614,911-7,838,927

Chr	BAC Name	BES	FISH	Sscrofa11.1 coordinates	USMARCv1.0 coordinates
8	PigE-134L21	CT126839.1; CT172501.1	8p	8:570,904-705,341	8:280,369,080-280,502,409
8	PigE-2N1	CT229915.1; CT229916.1	8p	8:819,717-958,131	8:137,599,822-137,737,820
8	PigE-118B21	CT048761.1; CT091504.1	8q	8:138,491,413-138,647,394	8:322,914-478,869
9	CH242-65G4	CU695192.2	9p	9:320,582-511,079	9:137,686,630-137,874,917
9	PigE-126O17	CT170583.1; CT057320.1	9p	9:443,462-603,022	9:137,594,779-137,754,110
9	PigE-242D8	CT123266.1; CT123265.1	9 mid	9:67,752,381-67,910,109	9:71,096,887-71,254,731
9	CH242-411M8	CT362997.1; CT468791.1	9q	9:139,180,446-139,338,710	9:168,756-327,007
10	CH242-451I23	CT369304.1; CT459538.1	10p	Unplaced scaffold: Contig2471	10:71,863,534-72,028,842
10	CH242-36D16	CT345373.1; CT186999.1	10q	10:55,422,866-55,600,351	10:15,300,371-15,480,359
10	CH242-517L16	FP325295.2	10q	10:55,609,778-55,800,022	10:15,098,969-15,290,916
11	PigE-199B10	CT272693.1	11p	11:135,233-297,713	11:79,101,520-79,264,254
11	PigE-232N19	CT193346.1	11p	11:290,540-291,222	11:79,108,017-79,108,697
11	PigE-211E21	CT044498.1; CT044499.1	11p	11:1,584,043-1,743,425	11:77,663,220-77,822.434
11	CH242-239O11	CT146353.1; CT286242.1	11q	11:78,888,491-79,057,526	11:827,483-996,382
12	PigE-253K5	CT081057.1; CT204391.1	12p	12:324,614-524,015	12:3,288-206,400
12	PigE-124G15	CT056668.1; CT092177.1	12q	12:60,846,540-60,990,610	12:58,746,918-58,890,342
13	PigE-197C11	CT271598.1; CT271599.1	13p	13:556,804-694,010	13:204,579,401-204,716,338
13	PigE-179J15	CT124924.1; CT124925.1	13q	13:205,856,740-206,006,912	13:3,005,553-3,154,893
14	PigE-137C12	FP340551.3	14p	14:17,423-156,591	14:140,940,126-140,804,938
14	PigE-167E18	CT089616.1; CT089617.1	14q	14:141,407,495-141,435,234	14:98,899-125,652
15	PigE-90C11	CT190903.1; CT190904.1	15p	15:3,442,144-3,596,666	15:139,733,189-139,886,921
15	PigE-108N22	CT073138.1; CT046453.1	15 mid	15:56,903,229-57,028,679	no match
15	CH242-170N3	FP236135.2	15q	15:139,616,279-139,784,756	15:3,511,408-3,588,855
16	PigE-90L22	CT191132.1; CT113297.1	16p	16:109,696-235,547	16:87,402-212,531
16	PigE-124C22	CT056551.1; CT056550.1	16p	16:117,329-308,428	16:94,873-287,243
16	CH242-4G9	CT041970.1; CT041969.1	16p	16:141,557-324,802	16:118,753-303,587
16	PigE-173H6	CT123878.1; CT123877.1	16p	16:167,106-299,570	16:144,276-278,432
16	PigE-149F10	CT088298.1; CT153977.1	16p	16:596,671-782,524	16:78,918,129-79,108,868
16	CH242-42L16	CT347302.1; CT347303.1	16q	16:79,097,179-79,303,695	16:878,687-1,085,418
17	CH242-70L7	CT077340.1; CT077341.1	17p	17:545,995-673,770	17:464,378-592,438
17	PigE-190G24	CT126644.1; CT096362.1	17p	17:515,422-707,787	17:433,829-626,496
17	CH242-243H19	CT321876.1; CT321877.1	17q	17:61,760-582-61,937,945	17:62,450,941-62,628,249

Chr	BAC Name	BES	FISH	Sscrofa11.1 coordinates	USMARCv1.0 coordinates
18	PigE-253N22	CT081116.1; CT204433.1	18p	18:1,616,389-1,751,286	18:1,565,719-1,700,920
18	PigE-202I11	CT042866.1; CT254626.1	18q	18:55,539,630-55,700,409	18:55,320,418-55,481,057
X	CH242-447L20	CT377508.1; CT467360.1	Xp	X:505,086-692,549	no match
X	CH242-156O11	FP074895.7	Xp + Yp	X:6,337,709 6,584,993	X:7,588,110-7,597,109
X	CH242-19N1	CU856094.8	Xp	X:6,705,194-6,834,183	X:7,588,110-7,715,932
X	CH242-305A15	CU861979.13	Xq	X:125,384,028-125,529,813	X:126,150,718-126,296,945
Y	CH242-156O11	FP074895.7	Xp + Yp	Y:4,744,231-4,791,971	Y:32,909,634-32,923,401

Table S5. Alignment of Radiation Hybrid maps and genome assemblies.

	Assembly		
	Sscrofa10.2	USMARCv1.0	Sscrofa11.1
% aligned	96.79	98.58	99.57
% within 1 place of expected order	73.67	76.44	76.29
% within 2 places of expected order	87.74	90.51	90.55
% within 3 places of expected order	92.88	95.36	95.38
% within 4 places of expected order	95.50	97.69	97.75
% within 5 places of expected order	96.96	98.64	98.70

All of the SNP markers from the Radiation Hybrid maps were ranked based on their RH map positions, sorted by the position on the genomes. Subsequently, the number of markers where the RH rank of the next SNP is +/- 1 position (or more as labelled) were counted and expressed as a percentage of the total number of markers (38,379).

Table S6. Assembyltics comparisons, assembly statistics. Assembly statistics* for pig genome assemblies subject to Assemblytics analyses

Assembly	Accession	Total (bp)(ungapped)	Scaffolds	Scaffold N50	Contigs	Contig N50
**Sscrofa11.1**	GCA_000003025.6	2,472,047,747	706	88,231,837	1,118	48,231,277
**USMARCv1.0**	GCA_002844635.1	2,623,130,238	14,818	131,458,098	14,818	6,372,407
**Bamei**	GCA_001700235.1	2,433,636,520	129,335	1,529,027	187,466	70,893
**Berkshire**	GCA_001700575.1	2,414,739,650	94,468	1,655,397	137,661	94,651
**Hampshire**	GCA_001700165.1	2,418,011,428	82,206	1,550,023	122,452	102,417
**Jinhua**	GCA_001700295.1	2,433,032,022	115,554	1,478,908	158,796	95,227
**Landrace**	GCA_001700215.1	2,420,570,845	94,659	1,407,841	141,909	88,142
**LargeWhite**	GCA_001700135.1	2,430,896,979	102,342	2,441,555	150,742	88,831
**Meishan**	GCA_001700195.1	2,438,814,343	133,833	1,248,180	201,146	63,263
**Pietrain**	GCA_001700255.1	2,415,062,022	88,436	1,663,542	139,497	80,611
**Rongchang**	GCA_001700155.1	2,429,730,895	120,246	2,325,000	173,508	79,093
**Tibetan**	GCA_000472085.2	2,379,878,366	72,068	861,885	148,234	57,199
**Wuzhishan**	GCA_000325925.2	2,453,484,489	137,577	5,853,977	272,163	31,939

* source NCBI Assembly

Results and output files from the Assemblytics analyses can be accessed in the GigaScience Database. http://dx.doi.org/10.5524/100732 [79]

Table S7. BUSCO results. BUSCO statistics, BUSCOv2 (OrthoDBv9).

	Pig Sscrofa10.2	Pig Sscrofa11.1	Pig USMARCv1.0	Human GRCh38p5	Mouse GRCm39p3
**Complete BUSCOs**	80.9%	93.8%	93.1%	94.9%	95.2%
**Complete and single-copy BUSCOs**	80.2%	93.3%	92.6%	94.1%	91.6%
**Complete and duplicated BUSCOs**	0.7%	0.5%	0.5%	0.8%	3.6%
**Fragmented BUSCOs**	8.2%	3.5%	3.5%	2.5%	2.3%
**Missing BUSCOs**	10.9%	2.7%	3.4%	2.6%	2.5%
**Total BUSCO groups searched**	4,104	4,104	4,104	4,104	4,104

Table S8. Annotation statistics. Comparison of Ensembl and NCBI annotation of Sscrofa11.1.

**Ensembl**		**NCBI**			
			missing (relative location)		
		in common	(intragenic)	(intergenic)	other
Protein-coding	21,301	17,676	468	2,617	[1]540
Non-coding	8,971	1,700	627	6,346	[2]298
Pseudogenes	1,626	134	4	1,193	[3]295
**NCBI**		**Ensembl**			
			missing (relative location)		
		in common	(intragenic)	(intergenic)	other
Protein-coding	20,790	17,676	140	2,522	[4]452
Non-coding	6,460	1,700	100	4,433	[5]227
Pseudogenes	3,084	134	4	2,884	[6]62

[1] total of 540 protein-coding genes of which 145 were annotated as non-coding by NCBI and the remaining 395 were annotated as pseudogenes by NCBI

[2] total of 298 non-coding genes of which 271 were annotated as protein-coding by NCBI and the remaining 27 were annotated as pseudogenes by NCBI

[3] total of 295 pseudogenes genes of which 281 were annotated as protein-coding by NCBI and the remaining 14 were annotated as non-coding by NCBI

[4] total of 452 protein-coding genes of which 222 were annotated as non-coding by Ensembl and the remaining 230 were annotated as pseudogenes by Ensembl

[5] total of 227 non-coding genes of which 215 were annotated as protein-coding by Ensembl and the remaining 12 were annotated as pseudogenes by Ensembl

[6] total of 62 pseudogenes genes of which 34 were annotated as protein-coding by Ensembl and the remaining 28 were annotated as non-coding by Ensembl

Table S9. Commercial SNP chip probes. SNP chip markers mapped to pig genome assemblies.

Assembly	Mapped / unmapped	AxiomHD	PorcineSNP60	GGP LD	80K
**Sscrofa10.2**	mapped	633,705	59,590	50,530	68,046
	unmapped	24,987	1,975	385	470
**Sscrofa11.1**	mapped	628,280	61,299	50,586	68,270
	ummapped	30,412	266	329	246
**USMARCv1.0**	mapped	618,771	60,692	50,042	67,604
	unmapped	39,921	873	873	912

Table S10. Tissue samples for PRJEB19386. Tissue samples characterised by Illumina short read RNA-Seq analyses.

Tissue	BioSample accession	alias	Animal	Sex
alveolar macrophages	SAMEA103886124	SUS_RI_DUR21-30	Duroc 21	female
alveolar macrophages	SAMEA103886168	SUS_RI_Pig 21_DUR_30	Duroc 21	female
alveolar macrophages	SAMEA103886137	SUS_RI_DUR22-60	Duroc 22	male
alveolar macrophages	SAMEA103886112	SUS_RI_Pig 22_DUR_60	Duroc 22	male
amygdala	SAMEA103886173	SUS_RI_R-Dur_23-08	Duroc 23	female
amygdala	SAMEA103886162	SUS_RI_Dur_24-C-S0	Duroc 24	male
brain, frontal lobe	SAMEA103886139	SUS_RI_R-Dur_23-01	Duroc 23	female
brain, frontal lobe	SAMEA103886156	SUS_RI_R-Dur_24-41	Duroc 24	male
brain stem	SAMEA103886128	SUS_RI_R-Dur_23-05	Duroc 23	female
brain stem	SAMEA103886129	SUS_RI_R-Dur_24-45	Duroc 24	male
caecum	SAMEA103886133	SUS_RI_DUR21-19	Duroc 21	female
caecum	SAMEA103886120	SUS_RI_DUR22-48	Duroc 22	male
caecum	SAMEA103886151	SUS_RI_Pig 22_DUR_48	Duroc 22	male
cerebellum	SAMEA103886116	SUS_RI_R-Dur_23-09	Duroc 23	female
cerebellum	SAMEA103886131	SUS_RI_R-Dur_24-49	Duroc 24	male
colon	SAMEA103886132	SUS_RI_Dur_23-21	Duroc 23	female
colon	SAMEA103886147	SUS_RI_Dur_24-61	Duroc 24	male
corpus callosum	SAMEA103886154	SUS_RI_R-Dur_23-10	Duroc 23	female
corpus callosum	SAMEA103886167	SUS_RI_R-Dur_24-50	Duroc 24	male
duodenum	SAMEA103886155	SUS_RI_Dur_23-22	Duroc 23	female
duodenum	SAMEA103886176	SUS_RI_Dur_24-62	Duroc 24	male
epididymis	SAMEA103886140	SUS_RI_DUR22-58	Duroc 22	male
hippocampus	SAMEA103886122	SUS_RI_Dur_23-B-S0	Duroc 23	female
hippocampus	SAMEA103886114	SUS_RI_R-Dur_24-51	Duroc 24	male
ileum	SAMEA103886163	SUS_RI_Dur_23-23	Duroc 23	female
ileum	SAMEA103886121	SUS_RI_Dur_24-63	Duroc 24	male
kidney cortex	SAMEA103886174	SUS_RI_DUR21-09	Duroc 21	female
kidney cortex	SAMEA103886153	SUS_RI_DUR22-39	Duroc 22	male
heart, left ventricle	SAMEA103886169	SUS_RI_DUR21-12	Duroc 21	female
heart, left ventricle	SAMEA103886172	SUS_RI_DUR22-43	Duroc 22	male
lymph node, mesenteric	SAMEA103886127	SUS_RI_DUR21-22	Duroc 21	female
lymph node, mesenteric	SAMEA103886115	SUS_RI_DUR22-51	Duroc 22	male
medulla oblongata	SAMEA103886135	SUS_RI_R-Dur_23-06	Duroc 23	female
medulla oblongata	SAMEA103886142	SUS_RI_R-Dur_24-46	Duroc 24	male

Tissue	BioSample accession	alias	Animal	Sex
occipital lobe	SAMEA103886158	SUS_RI_R-Dur_23-02	Duroc 23	female
occipital lobe	SAMEA103886177	SUS_RI_R-Dur_24-42	Duroc 24	male
omentum	SAMEA103886145	SUS_RI_DUR21-65	Duroc 21	female
omentum	SAMEA103886146	SUS_RI_DUR22-73	Duroc 22	male
penis	SAMEA103886166	SUS_RI_DUR22-59	Duroc 22	male
pituitary gland	SAMEA103886152	SUS_RI_Dur_23-14	Duroc 23	female
pituitary gland	SAMEA103886150	SUS_RI_Dur_24-54	Duroc 24	male
pituitary gland	SAMEA103886149	SUS_RI_DUR21-06	Duroc 21	female
pons	SAMEA103886159	SUS_RI_R-Dur_23-07	Duroc 23	female
pons	SAMEA103886164	SUS_RI_R-Dur_24-47	Duroc 24	male
skeletal muscle	SAMEA103886171	SUS_RI_DUR21-24	Duroc 21	female
skeletal muscle	SAMEA103886118	SUS_RI_DUR22-75	Duroc 22	male
spleen	SAMEA103886157	SUS_RI_DUR21-25	Duroc 21	female
spleen	SAMEA103886170	SUS_RI_DUR22-55	Duroc 22	male
stomach	SAMEA103886111	SUS_RI_Dur_23-24	Duroc 23	female
stomach	SAMEA103886134	SUS_RI_Dur_24-64	Duroc 24	male
thalamus	SAMEA103886136	SUS_RI_R-Dur_23-13	Duroc 23	female
thalamus	SAMEA103886160	SUS_RI_R-Dur_24-53	Duroc 24	male
tonsils	SAMEA103886125	SUS_RI_DUR22-56	Duroc 22	male
uterus	SAMEA103886126	SUS_RI_DUR21-27	Duroc 21	female

Figure S1. Predicted telomeres. Predicted locations of telomeres in the Sscrfoa11.1 assembly

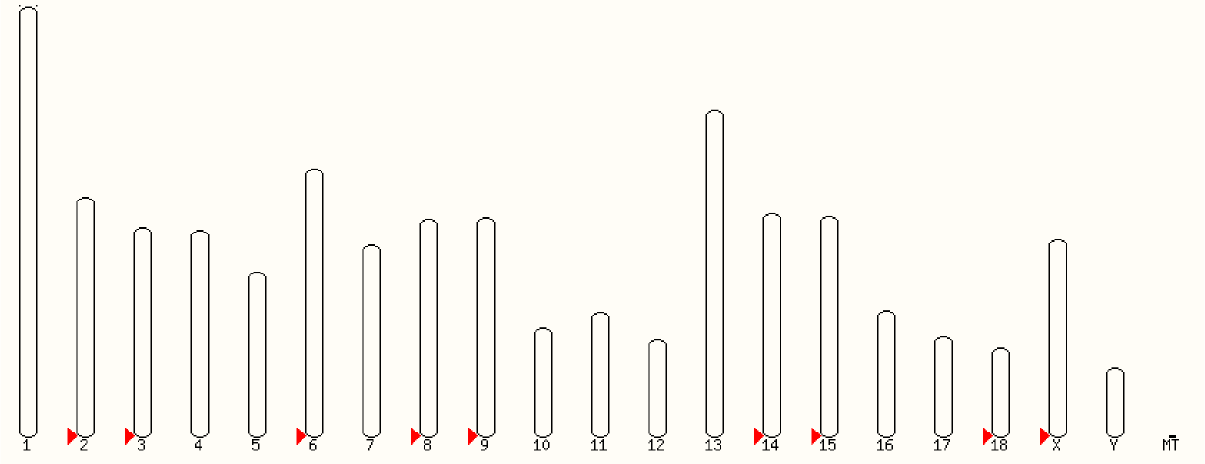

Figure S2. Predicted centromeres. Predicted centromere locations in the Sscrofa11.1 assembly.

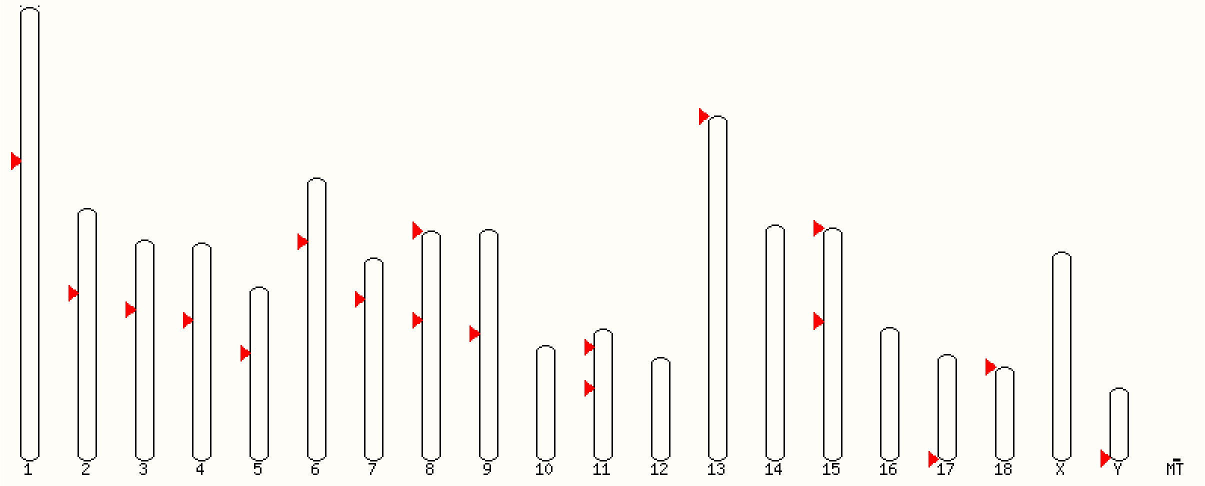

Figure S3. Fluorescent *in situ* hybridisation assignments.
a. SSC6 – p-telomeric end labelled with PigE-238J17, q-telomeric end labelled with CH242-510F2

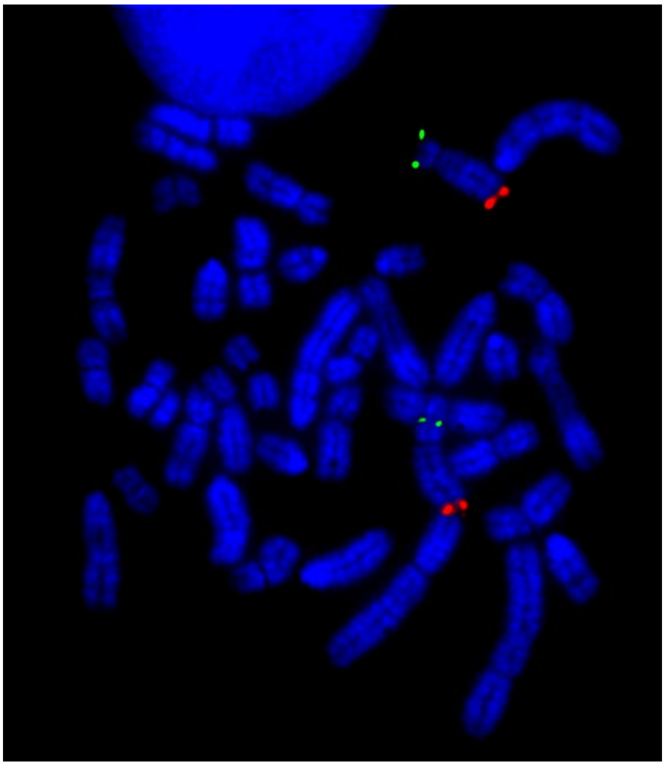

b. SSCX – p-telomeric end labelled with CH242-19N1, q-telomeric end labelled with CH242-305A15

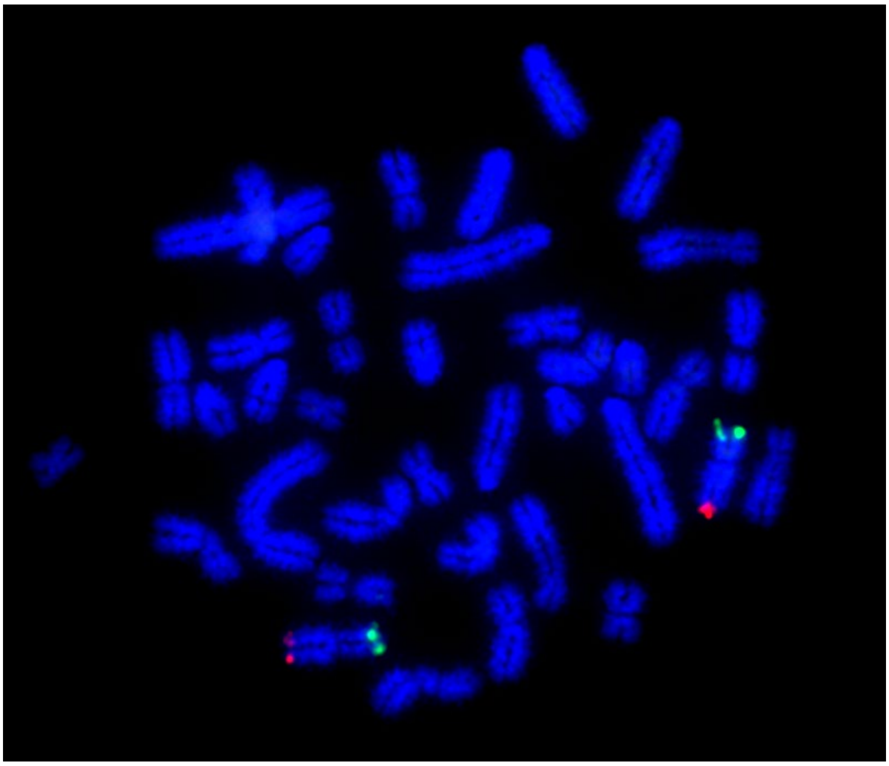

Fig. S4. Improvement in local order and orientation and reduction in redundancy. gEVAL screenshort showing the alignment of isogenic CH242 BAC end and WTSI_1005 fosmid end sequences with the Sscrofa10.2 (upper panel with pink bar on left hand side) and Sscrofa11.1 (lower panel). Red arrows indicate incorrect orientation of the paired end sequences, purple arrows are sequences which are present multiple times, green and orange arrows indicate the end sequences are correctly oriented. The distances between correctly oriented end sequences are as expected (green) or either greater or less than expected (orange) for the clone insert size for the fosmid or CH242 BAC libraries.

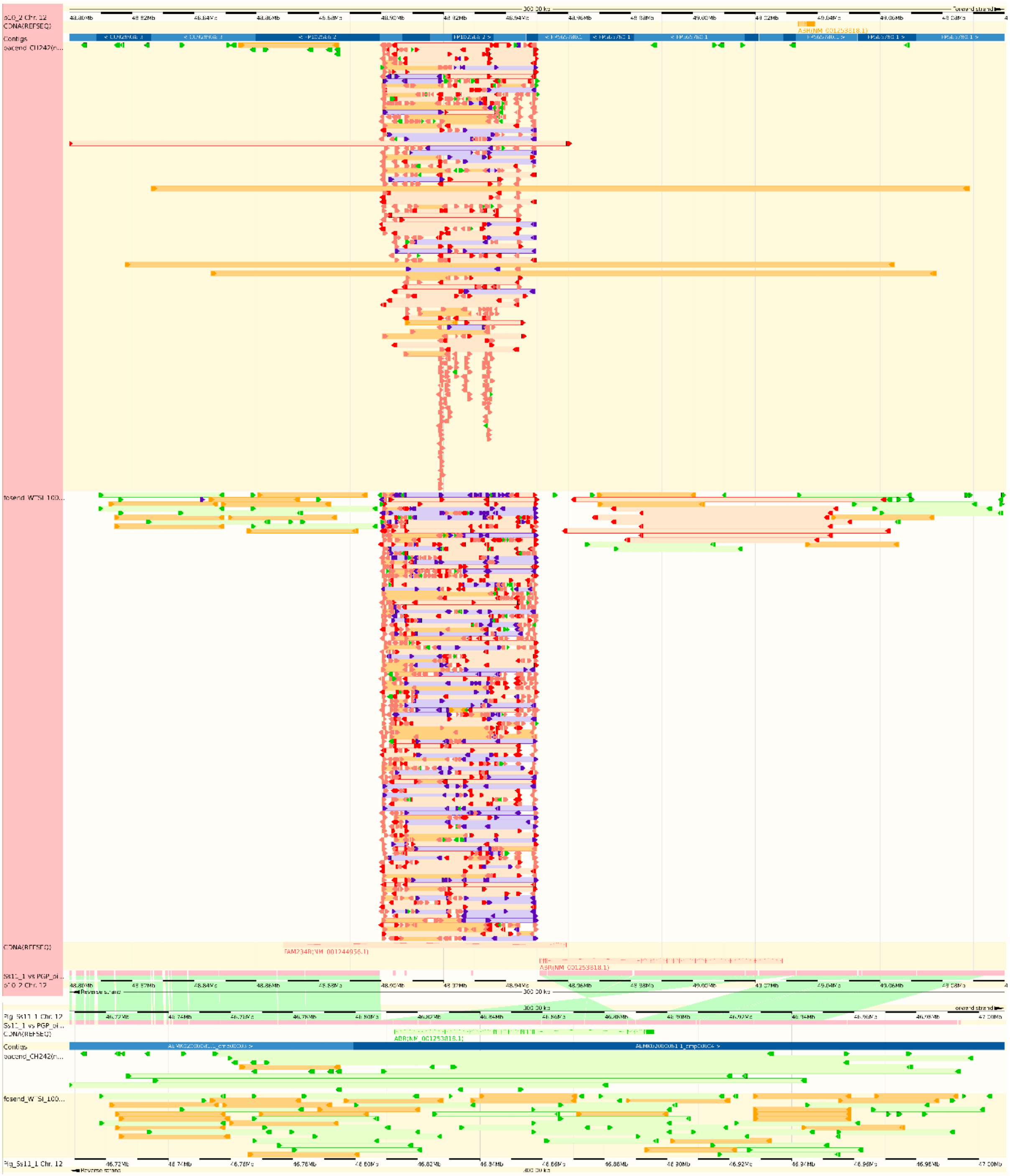

Fig. S5. Assembly comparisons in gEVAL (SSC15). Screenshot of gEVAL comparison of Sscrofa10.2, Sscrofa11.1 and USMARCv1.0 at *COL3A1*, *COL5A2* loci. In the new assembly (Sscrofa11.1, middle row marked with pink vertical block) an improved gene model for COL5A2 can be annotated; in the previous assembly (Sscrofa10.2, upper row) the order and orientation of sequence contigs within BAC clone CH242-40P12 (ENA: FP339585.2) are not resolved. There is good agreement between the Sscrofa11.1 (middle row) and the USMARCv1.0 (lower row) although the USMARCv1.0 assembly of SSC15 is inverted relative to Sscrofa11.1.

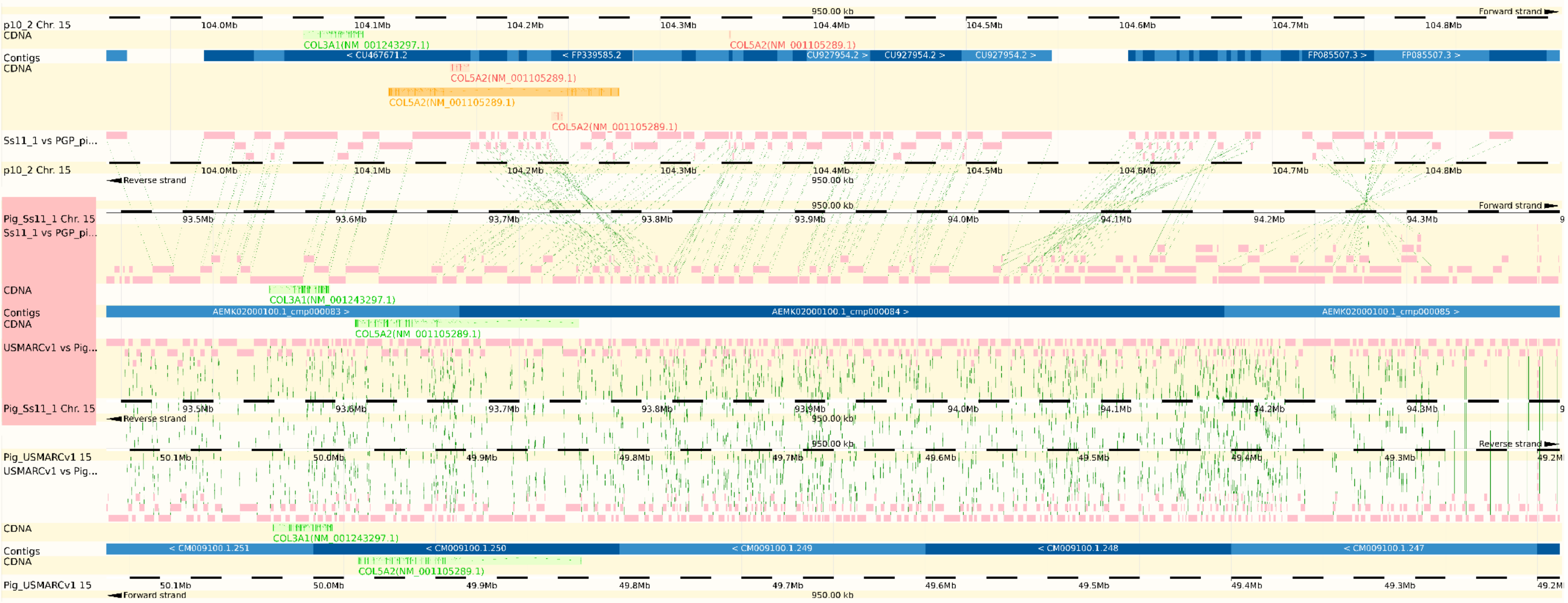

Fig. S6. Assembly comparisons in gEVAL (SSC5). Screenshot of gEVAL comparison of Sscrofa10.2, Sscrofa11.1 and USMARCv1.0 at the *KITLG* locus. The new assembly (Sscrofa11.1, middle row with pink vertical block at left hand side) resolves the sequences encoding *KITLG* which were split across two small scaffolds in Sscrofa10.2 (upper row). Although there is good agreement between Sscrofa11.1 (middle row) and USMARCv1.0 (lower row) assemblies in the right hand half of the region on SSC5 above, there is additional sequence present in the Sscrofa11.1 assembly between *DUSP6* and *KITLG*, the gene model for *KITLG* appears incomplete in the USMARCv1.0 assembly. Again the USMARCv1.0 is inverted relative to Sscrofa11.1.

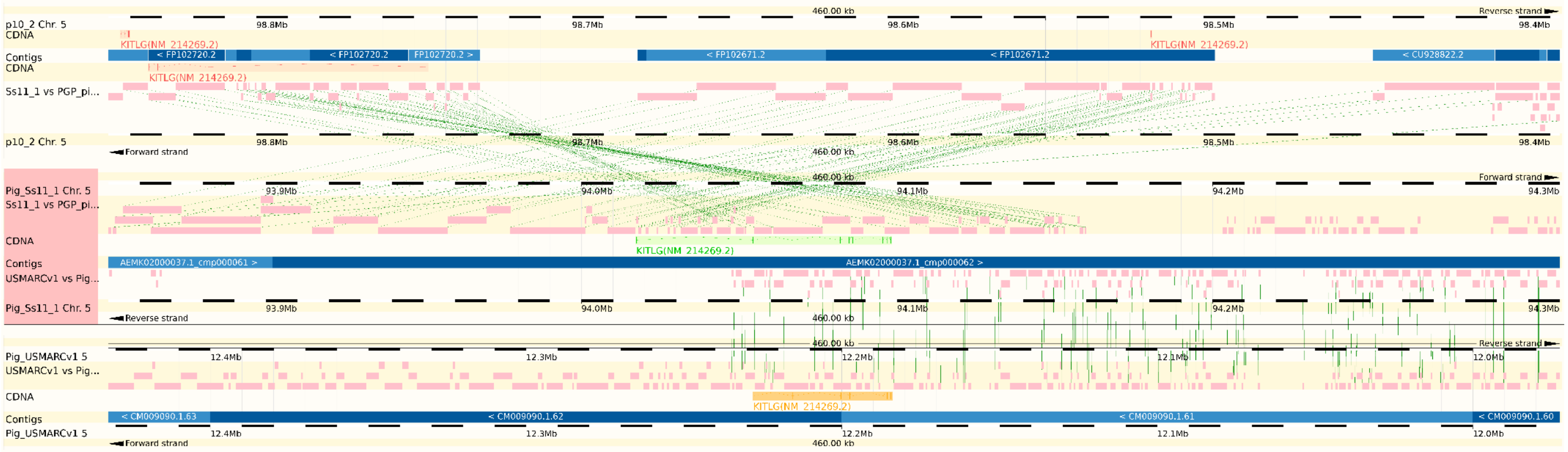

Fig. S7. Assembly comparisons in gEVAL (SSC18). Screenshot of gEVAL comparison of Sscrofa10.2, Sscrofa11.1 and USMARCv1.0 across the *ST7*, *CAPZA2* and *MET* loci on SSC18. The new assembly (Sscrofa11.1, middle row with pink block at left hand side) resolves the coding sequences for i) *ST7* that were previously split across two small scaffolds; *CAPZA2* that was similarly split across two small scaffolds; and iii) the *MET* sequences that were previously split as a result of an error in the orientation of the sequence drawn from BAC clone CH242-385N7 (ENA: CU633583.14) with respect to the sequence from BAC clone CH242-150K23 (ENA: CU694675.2) that harbours parts of the *MET* locus. This error in the incorporation of the CH242-385N7 (ENA: CU633583.14) in the Sscrofa10.2 assembly (upper row) is particularly unfortunate as this BAC had been sequenced to finish quality. There is good agreement between the Sscrofa11.1 (middle row) and USMARCv1.0 (lower row) assemblies with both SSC18 assemblies also being in the same orientation.

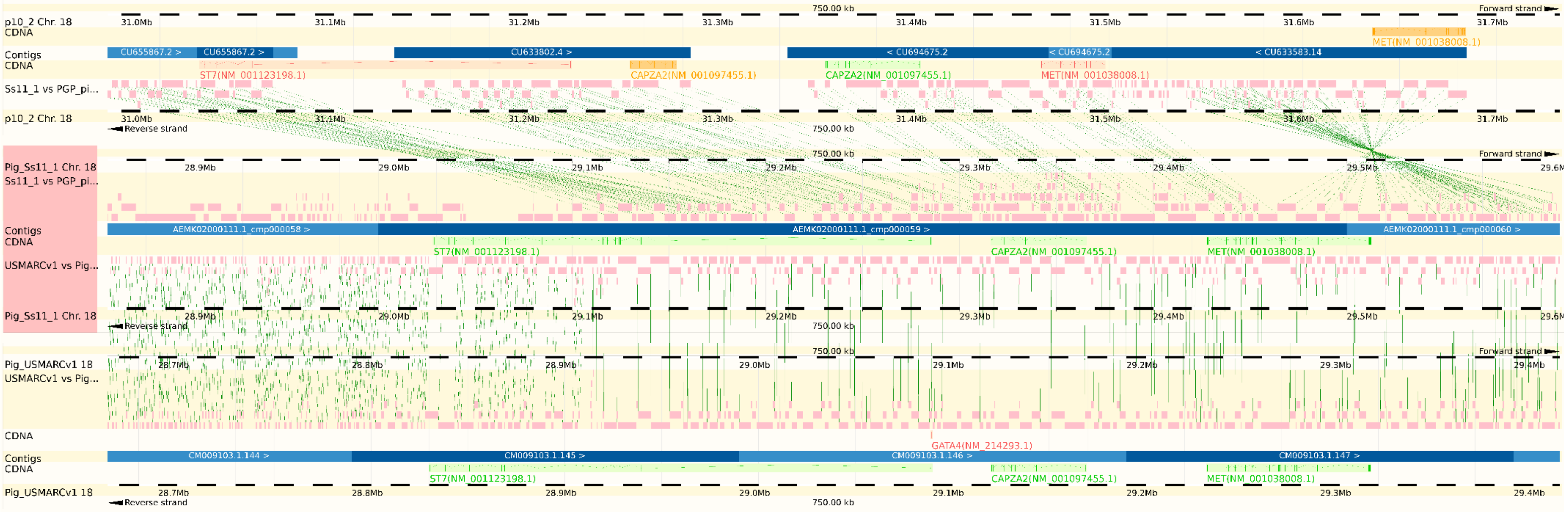

Fig. S8. Order and orientation of SSC18 assemblies. A. alignment of Sscrofa11.1 and USMARCv1.0 assemblies of SSC18; **B.** alignment of Sscrofa11.1 and radiation hybrid map (RH2); **C.** alignment of USMARCv1.0 and radiation hybrid map (RH2). The matches shown in the grey zone at the top of each plot of the Sscrofa11.1 versus USMARCv1.0 alignments probably represent a mix of repetitive sequences and matches to the unplaced scaffolds in the USMARCv1.0 assembly.

A. **B.** **C.**

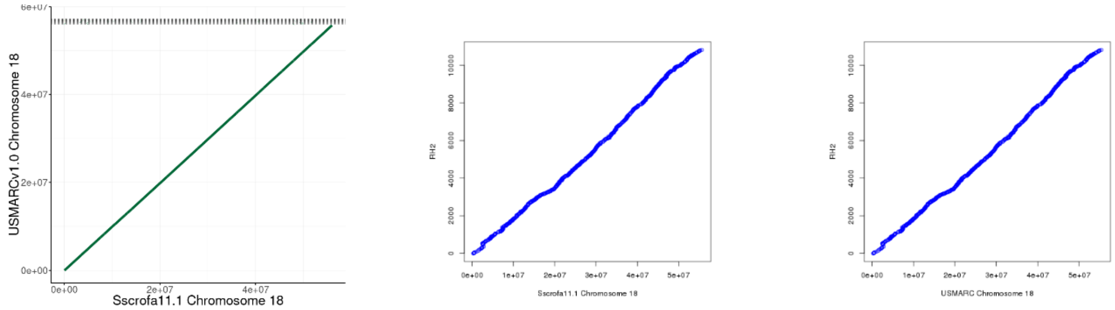

Fig. S9. **Order and orientation of SSC7 assemblies. A.** alignment of Sscrofa11.1 and USMARCv1.0 assemblies of SSC7; **B.** alignment of Sscrofa11.1 and radiation hybrid map (RH2); **C.** alignment of USMARCv1.0 and radiation hybrid map (RH2). The matches shown in the grey zone at the top of each plot of the Sscrofa11.1 versus USMARCv1.0 alignments probably represent a mix of repetitive sequences and matches to the unplaced scaffolds in the USMARCv1.0 assembly.

A. B. C.

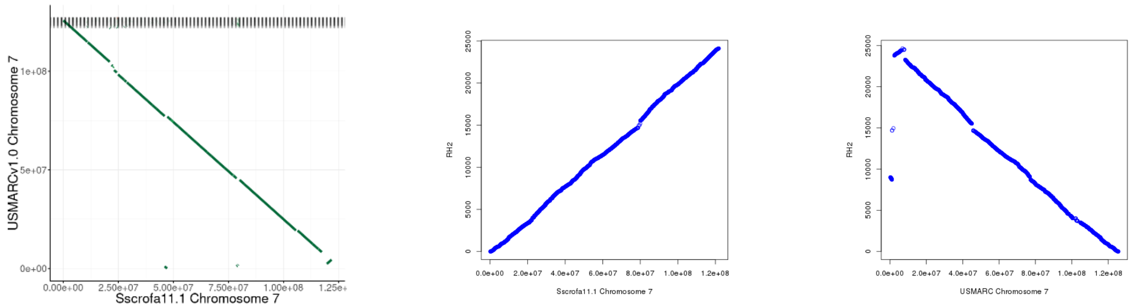

Fig. S10. Order and orientation of SSC8 assemblies. A. alignment of Sscrofa11.1 and USMARCv1.0 assemblies of SSC8; **B.** alignment of Sscrofa11.1 and radiation hybrid map (RH2); **C.** alignment of USMARCv1.0 and radiation hybrid map (RH2). The matches shown in the grey zone at the top of each plot of the Sscrofa11.1 versus USMARCv1.0 alignments probably represent a mix of repetitive sequences and matches to the unplaced scaffolds in the USMARCv1.0 assembly.

A. **B.** **C.**

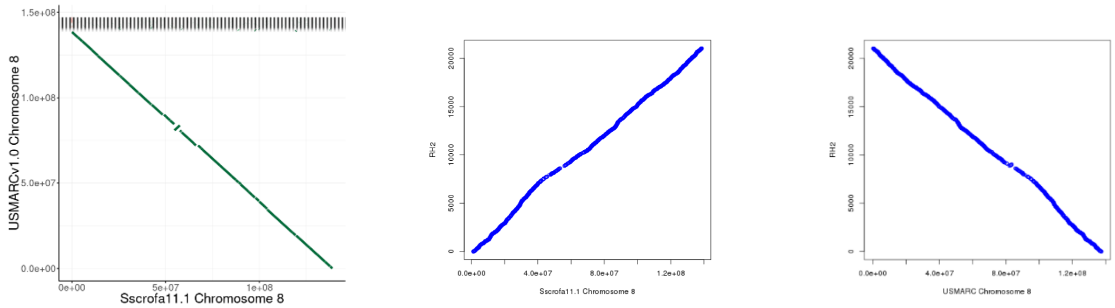

Fig. S11. Assembly alignments. Alignment of Sscrofa11.1 and USMARCv1.0 assemblies after correcting inversions of USMARCv1.0 chromosome scaffolds

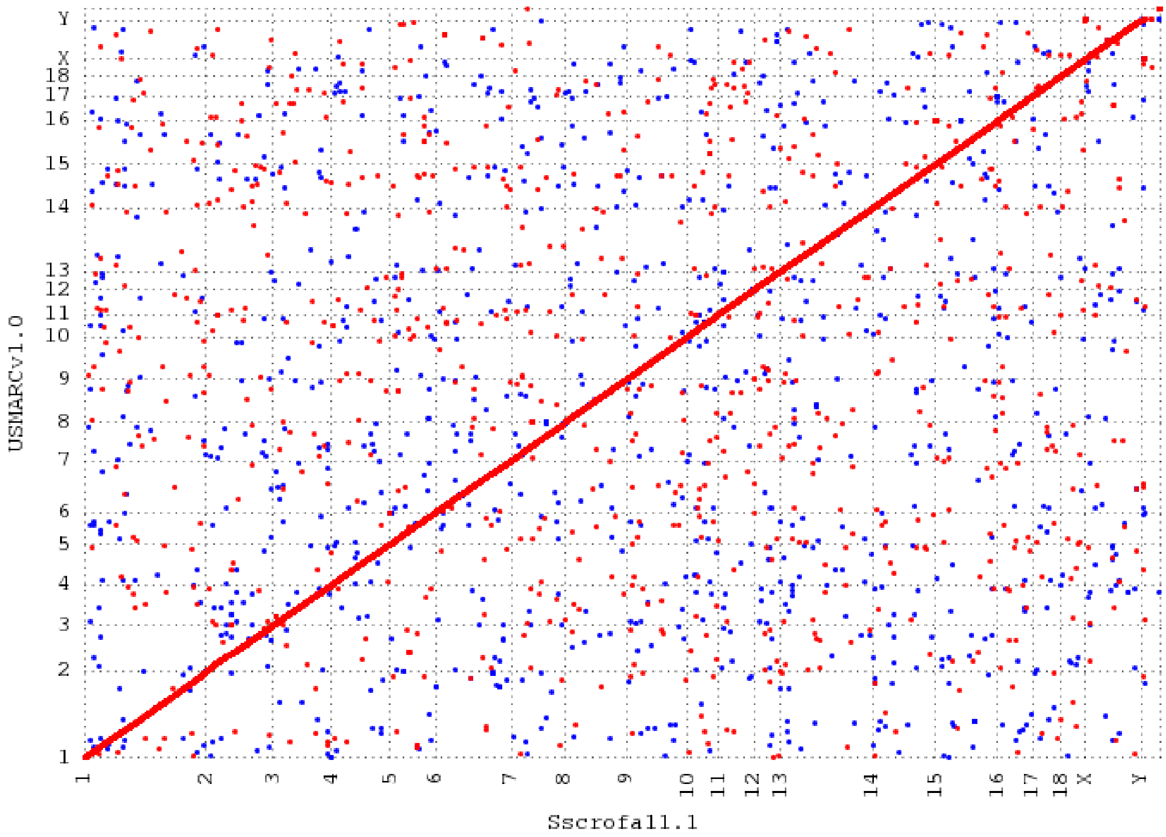

Figure S12a. Assemblytics results. Assemblytics comparison of Sscrofa11.1 (query) against the Sscrofa10.2 (reference) i). (left hand panel) variants from 50 to 500 bp; ii). (right hand panel) variants from 500 to 10,000 bp.

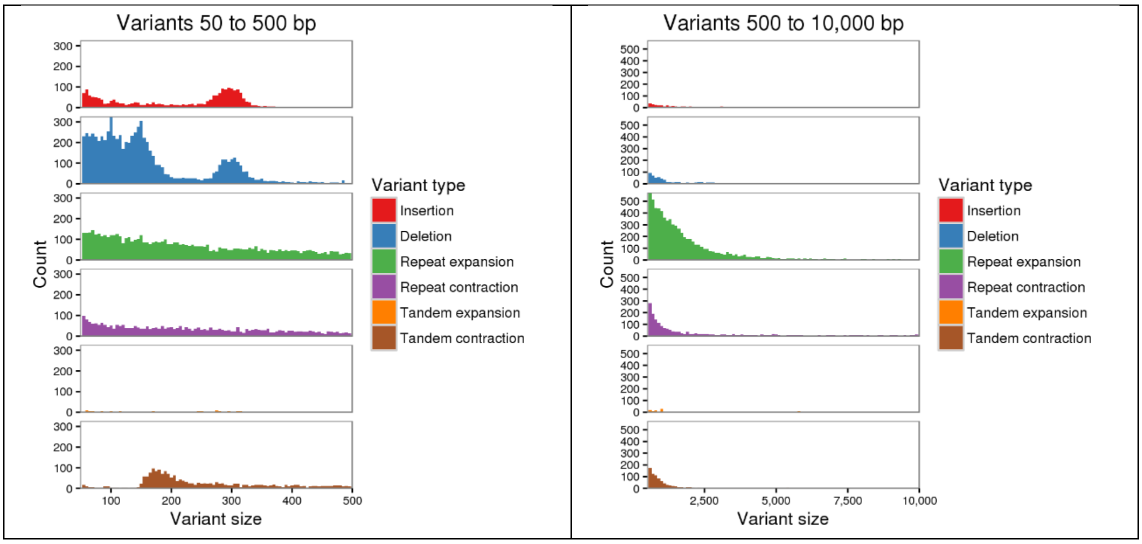

Figure S12b. Assemblytics results. Assemblytics comparison of USMARCv1.0 (query) against the Sscrofa10.2 (reference) i). (left hand panel) variants from 50 to 500 bp; ii). (right hand panel) variants from 500 to 10,000 bp.

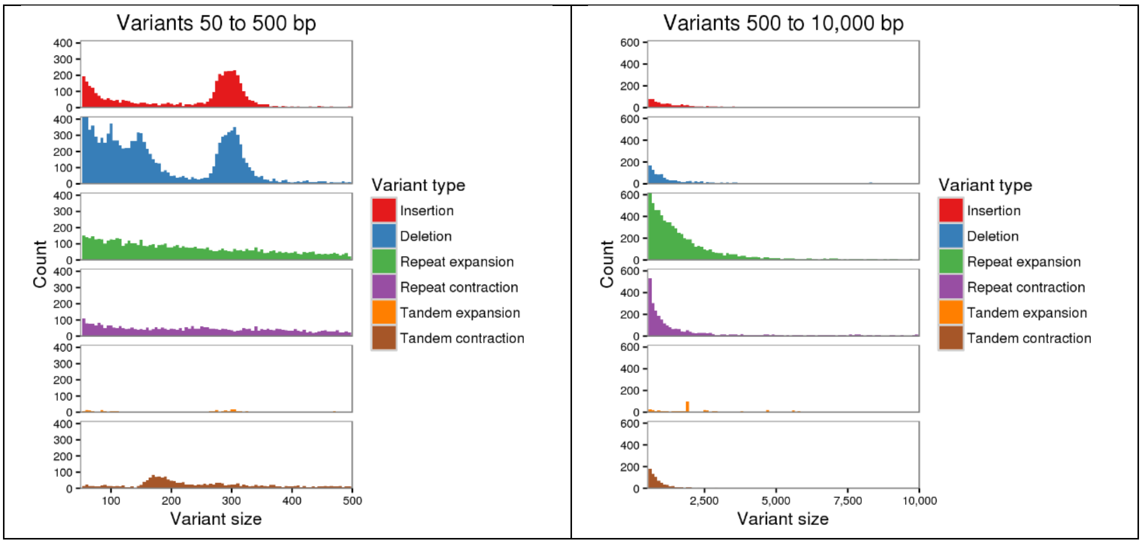

Figure S12c. Assemblytics results. Assemblytics comparison of USMARCv1.0 (query) against the Sscrofa11.1 (reference) i). (left hand panel) variants from 50 to 500 bp; ii). (right hand panel) variants from 500 to 10,000 bp.

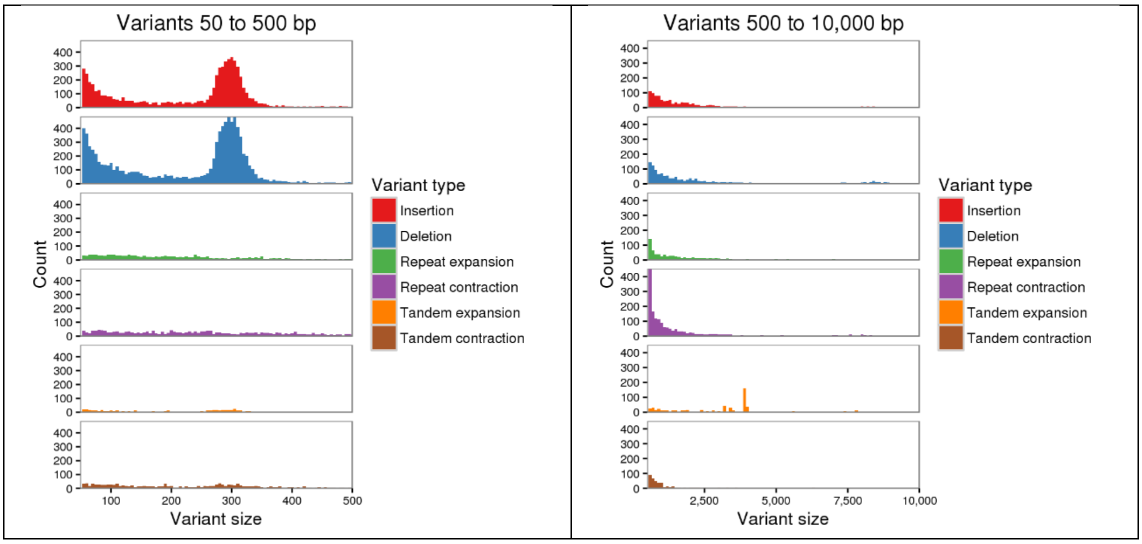

Figure S13. Counts of repetitive elements in four pig assemblies. Counts are given for repeat classes for which percent divergence was less than 40% and mapped length was above 70% relative to the RepBase database entries.

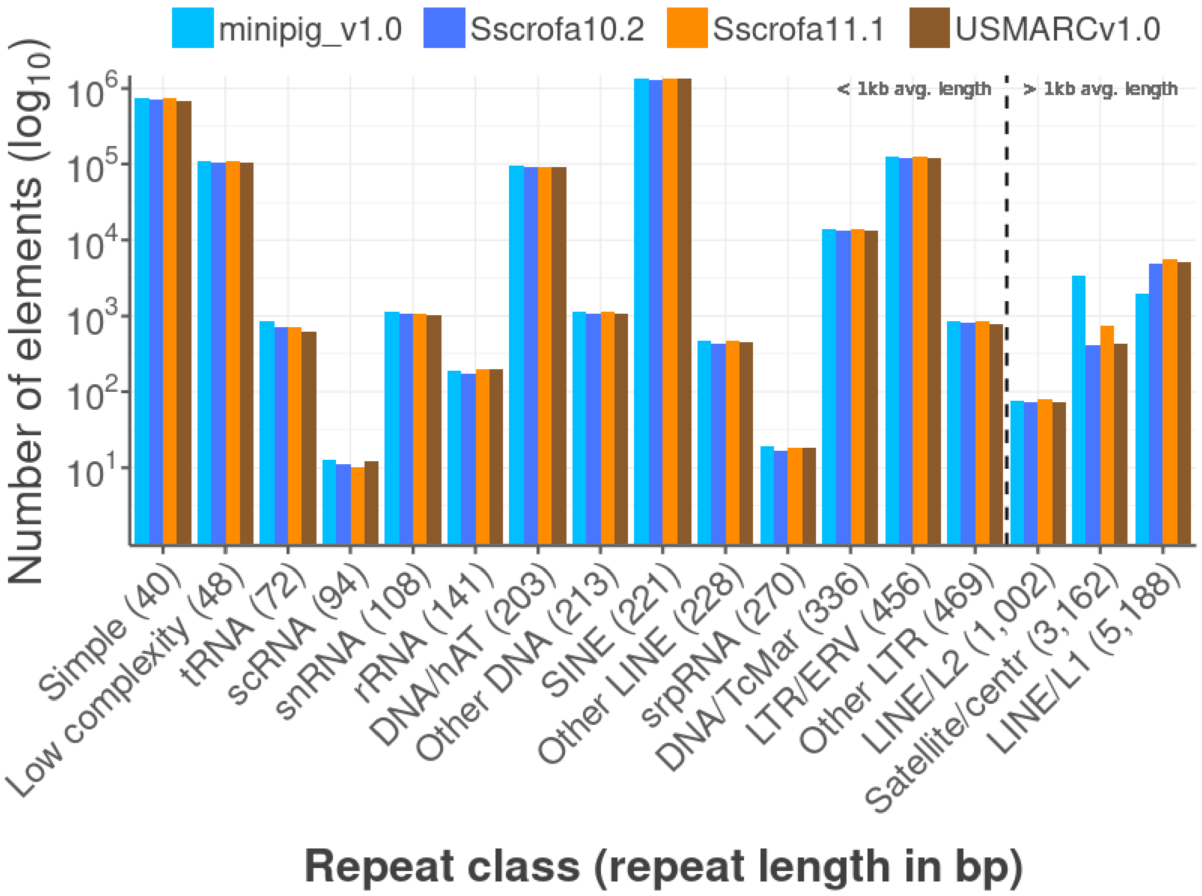

Figure S14. Average mapped length of repetitive elements in four pig genomes.

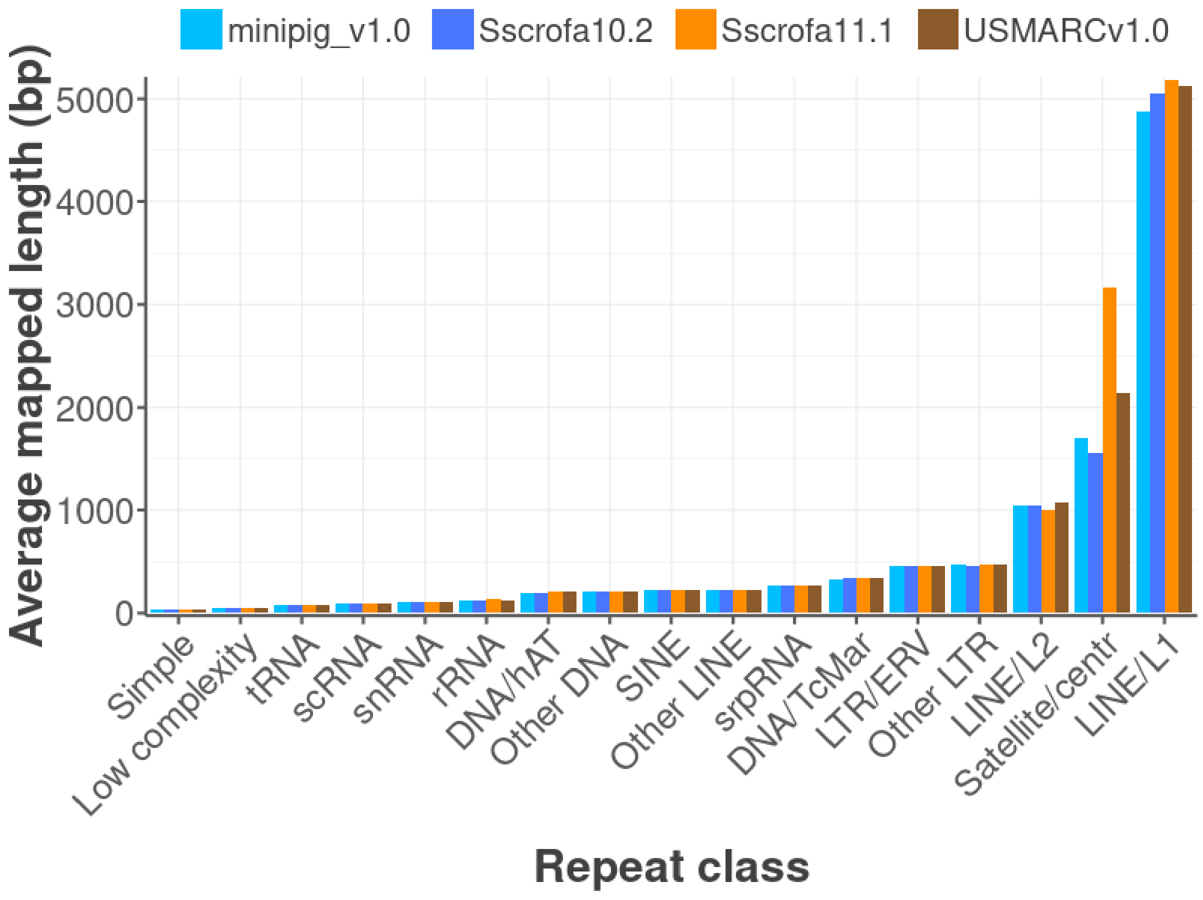

Figure S15. Assembly SNP rank concordance versus reported chromosomal location.

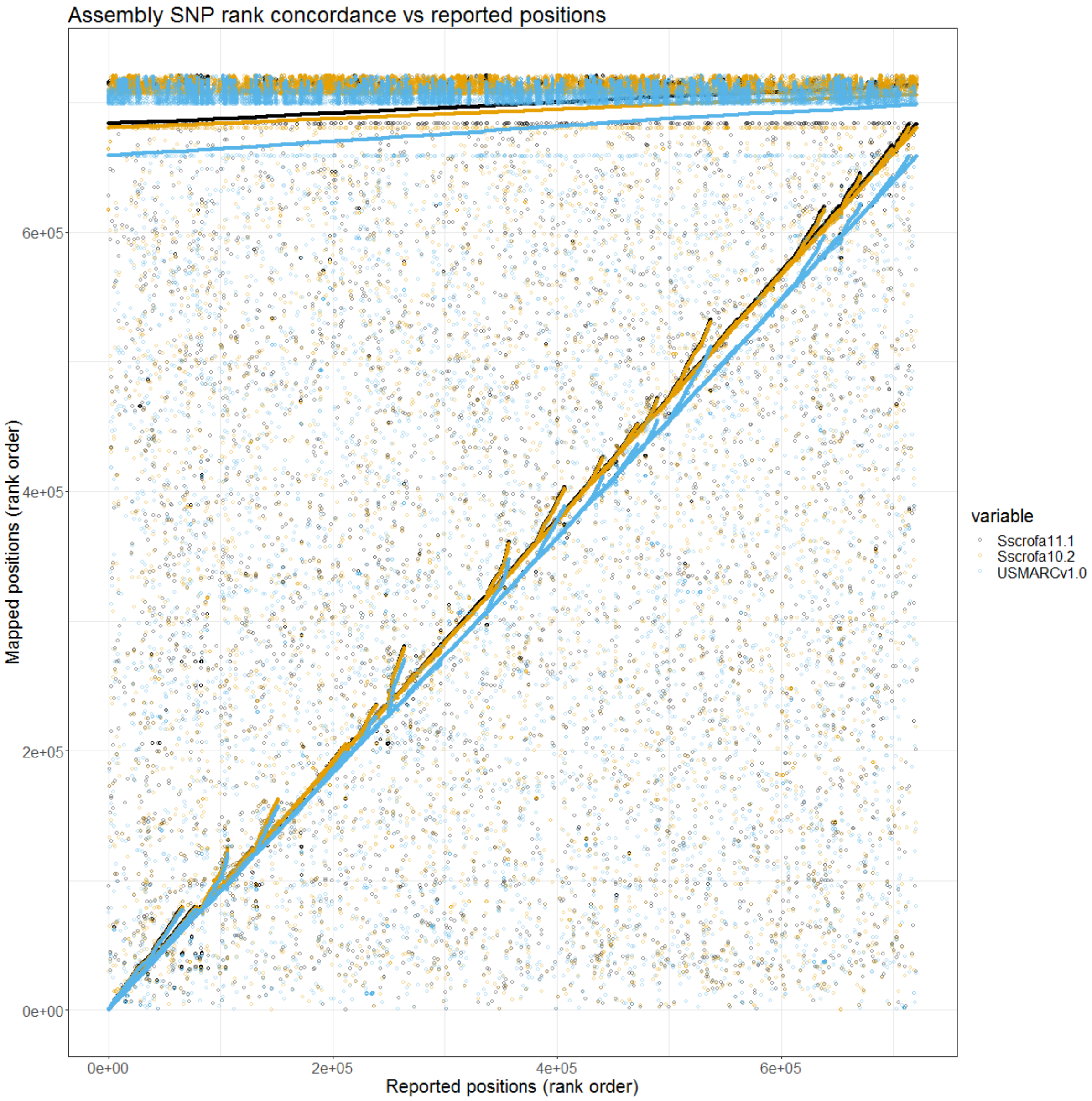

Supplement: giaa051_Supplemental_Files [file giaa051_supplemental_files.zip › Pig_genomes_suppl_10062020.pdf]
