# Supplementary material for: An improved pig reference genome sequence to enable pig genetics and genomics research
Source: Gigascience. 2020 Jun 16;9(6):giaa051. doi: 10.1093/gigascience/giaa051 (PMC7448572; doi:10.1093/gigascience/giaa051)
Supplement: giaa051_Response_to_Reviewer_Comments_Original_Submission [file giaa051_response_to_reviewer_comments_original_submission.pdf]

Author's Response To Reviewer Comments

Close

Reviewer reports:
Reviewer #2: The authors present us with two high-quality genome assemblies for the pig. In addition to the regular assembly procedure to obtain the two assemblies, they have made great efforts to check the accuracies of both using lots of other datasets, including FISH, radiation hybrid map, BAC clones. I only have several minor concerns as follows:
The authors annotated both the genomes using full-length transcriptome data from a single individual. I wonder whether you have any specific filtering step to avoid incorrect annotations, as the differential expression (both expression level and alternative splicing) may contribute to their phenotypic variances.
Line 180 - 190, the authors may want to explain more on the definition of low quality and low coverage regions, e.g. What're your criteria? Besides, please provide statistics of GC content for those remaining LQLC regions to show your points of view better.
For the assembly of USMARC, the authors mentioned that " The resulting assemblies were compared and the Celera Assembler result was selected based on better agreement with a Dovetail Chicago® library," it is better to explain more on your definition for the "better agreement".
Line 235 - 245, identify heterozygous structure variances using long reads can check whether the incongruencies between the v11.1 and v10.2 derived from innate differences between two haploids.

Responses
The authors annotated both the genomes using full-length transcriptome data from a single individual. I wonder whether you have any specific filtering step to avoid incorrect annotations, as the differential expression (both expression level and alternative splicing) may contribute to their phenotypic variances.
Whilst long read transcriptome data from one individual (i.e. MARC1423004 that was the source of DNA for the USMARCv1.0 assembly) was used to annotate the assemblies, short read RNA-seq data from this pig and four Duroc pigs (PRJEB19386, Duroc 21, Duroc 22, Duroc 23 & Duroc 24; see Table S9) was also used by the Ensembl Genebuild team. The NCBI annotation used further short read RNA-Seq data. The Ensembl annotation pipeline, including the filtering steps, is described in the Supplementary materials. There is good agreement between the Ensembl and NCBI annotation. Thus, we are confident that incorrect annotations have been minimised. Significantly more alternative transcripts have been captured in the annotation of the new assemblies. As noted in the manuscript information on expression levels for the Duroc pigs can be accessed through links from Ensembl genes to the EBI Gene Expression Database.
Line 180 - 190, the authors may want to explain more on the definition of low quality and low coverage regions, e.g. What're your criteria? Besides, please provide statistics of GC content for those remaining LQLC regions to show your points of view better.
The low coverage and low quality regions are as described in https://doi.org/10.3389/fgene.2015.00338. Briefly, Illumina data was mapped to the assembly, filtered to remove multimappers and the coverage over 1000bp windows was calculated. The coverage for each window was normalised for GC content. Regions deemed LQ were windows that had coverage more than 2 std above the median after normalisation for GC content, where the count of reads that were not properly paired was 2 std above the mean, or the number of reads with unexpectedly large/small insert sizes was 2 std above the mean. The LC regions were windows that had coverage more than 2 std below the median after normalisation for GC content. The LC regions are given separately because they are more likely to include, for example, repetitive regions where multimappers are more likely to have been, or regions of extreme GC content which had such low coverage to begin with that the normalisation was insufficient to correct this. We therefore have more confidence in the LQ regions representing true misassemblies/structural variation than the LC regions although the drop in LC regions from 10.2 to 11.1 does suggest that for the former assembly many of these were true misassemblies. This description has not been included in the manuscript as it is described fully in the cited paper, however a brief explanation has been added as to what these categories include to make this clearer without having to read the other manuscript (line 182).
The average GC content of the regions was calculated at 61.6%, which indeed supports our suggestion that the remaining regions may relate more to biases in the sequencing technology than actual error,

and this has been added to the text on line 189.

Change line 182-183
From: “Alignments of Illumina sequence reads from the same female pig were used to identify regions of low quality (LQ) or low coverage (LC) (Table 2).”
To: “Alignments of Illumina sequence reads from the same female pig were used to identify regions of low quality (LQ; regions with high GC normalised coverage, prevalence of improperly paired reads and prevalence of reads with improper insert sizes) or low coverage (LC; regions with low GC normalised coverage) (Table 2).”
Change old line 187:
From “The remaining LQLC segments of Sscrofa11 may represent regions where short read coverage is low due to known systematic errors of the short read platform related to GC content, rather than deficiencies of the assembly.”
To “The remaining LQLC segments of Sscrofa11 have an average GC content of 61.6%. Thus, these regions may represent sequence where short read coverage is low due to the known systematic bias of the short read platform against extreme GC content sequences, rather than deficiencies of the assembly.”

For the assembly of USMARC, the authors mentioned that " The resulting assemblies were compared and the Celera Assembler result was selected based on better agreement with a Dovetail Chicago® library," it is better to explain more on your definition for the "better agreement".
The resulting assemblies were compared and the Celera Assembler result was selected based on a lower proportion of conflicting links between read pairs of a Dovetail Chicago library, with fewer suggested breaks in the contigs. The relevant sentence has now been modified.

Line 235 - 245, identify heterozygous structure variances using long reads can check whether the incongruencies between the v11.1 and v10.2 derived from innate differences between two haploids.
The very significant reductions in low quality and low coverage regions from Sscrofa10.2 to Sscrofa11.1 (see earlier comment) confirms that Sscrofa11.1 is a significantly better representation of the genome sequence of Duroc sow 2-14. The Sscrofa10.2 assembly was generated from sequences of individual BAC clones and for the region covered by any individual BAC clone captures only one of the two haplotypes for the region. The Sscrofa11.1 assembly was assembled from whole genome shotgun sequence data and switches between haplotypes are more difficult to detect. Thus, any analysis of the haplotypes captured in these two assemblies would be compromised by the differences in sequencing strategy and in quality between the two assemblies.
We have clarified the text to read:
“Both Sscrofa11.1 and USMARCv1.0 assemblies have more differences against Sscrofa10.2 (33,347 and 44,023 respectively) than against each other (28,733). This is despite the fact that Sscroffa11.1 and Sscrofa10.2 represent the same pig genome. While some differences between Sscrofa10.2 and Sscrofa11.1 may be due to differences in which haplotype has been captured in the assembly, the reduction in low quality and low coverage regions and the dramatic decrease in differences versus USMARCv1.0 leads us to conclude that the majority are improvements in the assembly of Sscrofa11.1. The differences between the Sscrofa11.1 and USMARCv1.0 will represent a mix of true structural differences and assembly errors that will require further research to resolve.”

Close
