# Supplementary material for: An improved pig reference genome sequence to enable pig genetics and genomics research
Source: Gigascience. 2020 Jun 16;9(6):giaa051. doi: 10.1093/gigascience/giaa051 (PMC7448572; doi:10.1093/gigascience/giaa051)
Supplement: giaa051_Response_to_Reviewer_Comments_Revision_1 [file giaa051_response_to_reviewer_comments_revision_1.pdf]

Author's Response To Reviewer Comments

Close

Reviewer reports:
Reviewer #1:
Mingzhou Li (Reviewer 1): The domestic pig is of enormous agricultural significance and valuable models for many human diseases. Nonetheless, the draft assembly of the reference pig genome (Sscrofa10.2) was incomplete (at least 8% of the sequence is estimated to be missing from the assembly) and limited its utility. The MS entitled "An improved pig reference genome sequence to enable pig genetics and genomics research" reported two annotated highly contiguous chromosome-level genome assemblies (i.e., Sscrofa11.1 and USMARCv1.0) and also presented annotation of a further 11 short read assemblies of representative pig breeds in Europe and Asia. Especially, the updated Sscrofa11.1 (Contig N50 = 48.23 Mb, scaffold N50 = 88.23 Mb,) is substantively superior than the former version of Sscrofa10.2 (Contig N50 = 69.50 Kb, scaffold N50 = 576.01 Kb). To the best of my knowledge, this high-quality assembly of the reference pig genome (Sscrofa11.1, released at Dec 2016) had been widely adapted by the pig genomics community.
I appreciate authors' significant efforts for the pig genomics community, which provide an unprecedented view of the genetic make-up of this important agricultural and biomedical model species. The quality of the presentation is excellent, the structure of the presentation is clear and there are a very small number of typographical errors. Overall the discussions and conclusions appear sound and objective.
Specific comments:
1) Lines 50-51 "The domestic pig (Sus scrofa) is important both as a food source and as a biomedical model with high anatomical and immunological similarity to humans".
It is well documented that, compared with rodent, pig is closely comparable to human in size, anatomy, physiology, metabolism, pathology and pharmacology. Why only highlight "immunological similarity" here? As well as in Line 72: "including responses to infectious diseases".
2) Line 123 "MARC1423004 which was a Duroc/Landrace/Yorkshire crossbred barrow (i.e. castrated male pig)" . Is it means the terminal crossbreeding system with three pig breeds, i.e., Duroc × (Landrace × Yorkshire) (DLY). I think the author should provide the accurate description.
3) Lines 220-221 "After correcting the orientation of these inverted scaffolds, there is good agreement between the USMARCv1.0 assembly and the RH map (Fig. 1b)." I suggest the author should provide the exact statistic number to support the statement of "good agreement".
4）Lines 286-287: "There were five genes that were present in the Iso-Seq data, but missing in the Sscrofa11.1 assembly.". I have not find the corresponding description of the method and the more detail results of the "identification of missing genes in the assembly" . I think the author should provide these essential information. Given the volume of information available, it is difficult to assess the methodology.
5) Lines 548-549: "haplotype resolved assemblies of a Meishan and White Composite F1 crossbred pig currently being sequenced." Same as my comment 2), the author should accurately provide description of the sample.

Responses
Specific comments:
1) Lines 50-51 "The domestic pig (Sus scrofa) is important both as a food source and as a biomedical model with high anatomical and immunological similarity to humans".
It is well documented that, compared with rodent, pig is closely comparable to human in size, anatomy, physiology, metabolism, pathology and pharmacology. Why only highlight "immunological similarity" here? As well as in Line 72: "including responses to infectious diseases".
We have changed this opening line of the abstract to:
"The domestic pig (Sus scrofa) is important both as a food source and as a biomedical model given its similarity in size, anatomy, physiology, metabolism, pathology and pharmacology to humans.” (lines 50-51)
We have changed this text in original lines 69-72 to:
In farmed animal species such as the domestic pig (Sus scrofa) genome sequences have been integral to

the discovery of molecular genetic variants and the development of single nucleotide polymorphism (SNP) chips [1] and enabled efforts to dissect the genetic control of complex traits, such as growth, feed conversion, body composition, reproduction, behaviour and responses to infectious diseases [2]. (lines 69-73).

2) Line 123 "MARC1423004 which was a Duroc/Landrace/Yorkshire crossbred barrow (i.e. castrated male pig)" . Is it means the terminal crossbreeding system with three pig breeds, i.e., Duroc × (Landrace × Yorkshire) (DLY). I think the author should provide the accurate description.

This statement has been replaced with the following : ” MARC1423004 which was a crossbred barrow (i.e. castrated male pig) from a composite population (approximately ½ Landrace, ¼ Duroc and ¼ Yorkshire) at the USDA Meat Animal Research Center.” (lines 124-125)

3) Lines 220-221 "After correcting the orientation of these inverted scaffolds, there is good agreement between the USMARCv1.0 assembly and the RH map (Fig. 1b)." I suggest the author should provide the exact statistic number to support the statement of "good agreement".

While the plots demonstrate visually good overall agreement between the RH maps and the assemblies, we have provided statistics showing the finer scale agreement (new Supplementary Table S5). We show the proportion of SNPs whose neighbours are adjacent in both the genome alignment and the RH map. The additional table is cited in the text as follows:

“After correcting the orientation of these inverted scaffolds, there is good agreement between the USMARCv1.0 assembly and the RH map [9] (Fig. 1b, Table S5).” (lines 224-225).

4）Lines 286-287: "There were five genes that were present in the Iso-Seq data, but missing in the Sscrofa11.1 assembly.". I have not find the corresponding description of the method and the more detail results of the "identification of missing genes in the assembly" . I think the author should provide these essential information. Given the volume of information available, it is difficult to assess the methodology.

The ‘missing genes’ were identified by the Cogent analysis as clearly described in the manuscript in the section headed “Completeness of the assemblies” (lines 268- 295). Each of the missing genes were supported by multiple lines of evidence: (1) there were two or more full-length transcript isoforms, often from multiple tissues, from the PacBio Iso-Seq data; (2) the Iso-Seq transcripts had a BLAST hit to other species that were used to identify the missing gene name as stated in lines 290-295

5) Lines 548-549: "haplotype resolved assemblies of a Meishan and White Composite F1 crossbred pig currently being sequenced." Same as my comment 2), the author should accurately provide description of the sample.

This pig is an F1 between a Meishan and a pig from the USDA MARC composite line (approximately ½ Landrace, ¼ Duroc and ¼ Yorkshire) as for MARC1423004. The text has been modified as follows:

(ii) haplotype resolved assemblies of a Meishan and White Composite F1 crossbred pig (i.e. the offspring of a Meishan sire and a White Composite dam that is approximately ½ Landrace, ¼ Duroc and ¼ Yorkshire) currently being sequenced. (lines 552-554)

Reviewer #2: The authors present us with two high-quality genome assemblies for the pig. In addition to the regular assembly procedure to obtain the two assemblies, they have made great efforts to check the accuracies of both using lots of other datasets, including FISH, radiation hybrid map, BAC clones. I only have several minor concerns as follows:

The authors annotated both the genomes using full-length transcriptome data from a single individual. I wonder whether you have any specific filtering step to avoid incorrect annotations, as the differential expression (both expression level and alternative splicing) may contribute to their phenotypic variances.

Line 180 - 190, the authors may want to explain more on the definition of low quality and low coverage regions, e.g. What're your criteria? Besides, please provide statistics of GC content for those remaining LQLC regions to show your points of view better.

For the assembly of USMARC, the authors mentioned that " The resulting assemblies were compared and the Celera Assembler result was selected based on better agreement with a Dovetail Chicago® library," it is better to explain more on your definition for the "better agreement".

Line 235 - 245, identify heterozygous structure variances using long reads can check whether the incongruencies between the v11.1 and v10.2 derived from innate differences between two haploids.

Responses

The authors annotated both the genomes using full-length transcriptome data from a single individual. I wonder whether you have any specific filtering step to avoid incorrect annotations, as the differential expression (both expression level and alternative splicing) may contribute to their phenotypic variances.

Whilst long read transcriptome data from one individual (i.e. MARC1423004 that was the source of DNA for the USMARCv1.0 assembly) was used to annotate the assemblies, short read RNA-seq data from this pig and four Duroc pigs (PRJEB19386, Duroc 21, Duroc 22, Duroc 23 & Duroc 24; see Table S9) was

also used by the Ensembl Genebuild team. The NCBI annotation used further short read RNA-Seq data. The Ensembl annotation pipeline, including the filtering steps, is described in the Supplementary materials. There is good agreement between the Ensembl and NCBI annotation. Thus, we are confident that incorrect annotations have been minimised. Significantly more alternative transcripts have been captured in the annotation of the new assemblies. As noted in the manuscript information on expression levels for the Duroc pigs can be accessed through links from Ensembl genes to the EBI Gene Expression Database.
Line 180 - 190, the authors may want to explain more on the definition of low quality and low coverage regions, e.g. What're your criteria? Besides, please provide statistics of GC content for those remaining LQLC regions to show your points of view better.
The low coverage and low quality regions are as described in https://doi.org/10.3389/fgene.2015.00338. Briefly, Illumina data was mapped to the assembly, filtered to remove multimappers and the coverage over 1000bp windows was calculated. The coverage for each window was normalised for GC content. Regions deemed LQ were windows that had coverage more than 2 std above the median after normalisation for GC content, where the count of reads that were not properly paired was 2 std above the mean, or the number of reads with unexpectedly large/small insert sizes was 2 std above the mean. The LC regions were windows that had coverage more than 2 std below the median after normalisation for GC content. The LC regions are given separately because they are more likely to include, for example, repetitive regions where multimappers are more likely to have been, or regions of extreme GC content which had such low coverage to begin with that the normalisation was insufficient to correct this. We therefore have more confidence in the LQ regions representing true misassemblies/structural variation than the LC regions although the drop in LC regions from 10.2 to 11.1 does suggest that for the former assembly many of these were true misassemblies. This description has not been included in the manuscript as it is described fully in the cited paper, however a brief explanation has been added as to what these categories include to make this clearer without having to read the other manuscript (line 182).
The average GC content of the regions was calculated at 61.6%, which indeed supports our suggestion that the remaining regions may relate more to biases in the sequencing technology than actual error, and this has been added to the text on line 189.

Change line 182-183
From: “Alignments of Illumina sequence reads from the same female pig were used to identify regions of low quality (LQ) or low coverage (LC) (Table 2).”
To: “Alignments of Illumina sequence reads from the same female pig were used to identify regions of low quality (LQ; regions with high GC normalised coverage, prevalence of improperly paired reads and prevalence of reads with improper insert sizes) or low coverage (LC; regions with low GC normalised coverage) (Table 2).”
Change old line 187:
From “The remaining LQLC segments of Sscrofa11 may represent regions where short read coverage is low due to known systematic errors of the short read platform related to GC content, rather than deficiencies of the assembly.”
To “The remaining LQLC segments of Sscrofa11 have an average GC content of 61.6%. Thus, these regions may represent sequence where short read coverage is low due to the known systematic bias of the short read platform against extreme GC content sequences, rather than deficiencies of the assembly.”

For the assembly of USMARC, the authors mentioned that " The resulting assemblies were compared and the Celera Assembler result was selected based on better agreement with a Dovetail Chicago® library," it is better to explain more on your definition for the "better agreement".
The resulting assemblies were compared and the Celera Assembler result was selected based on a lower proportion of conflicting links between read pairs of a Dovetail Chicago library, with fewer suggested breaks in the contigs. The relevant sentence has now been modified.

Line 235 - 245, identify heterozygous structure variances using long reads can check whether the incongruencies between the v11.1 and v10.2 derived from innate differences between two haploids.
The very significant reductions in low quality and low coverage regions from Sscrofa10.2 to Sscrofa11.1 (see earlier comment) confirms that Sscrofa11.1 is a significantly better representation of the genome sequence of Duroc sow 2-14. The Sscrofa10.2 assembly was generated from sequences of individual BAC clones and for the region covered by any individual BAC clone captures only one of the two haplotypes for the region. The Sscrofa11.1 assembly was assembled from whole genome shotgun sequence data and switches between haplotypes are more difficult to detect. Thus, any analysis of the

haplotypes captured in these two assemblies would be compromised by the differences in sequencing strategy and in quality between the two assemblies.
We have clarified the text to read:
“Both Sscrofa11.1 and USMARCv1.0 assemblies have more differences against Sscrofa10.2 (33,347 and 44,023 respectively) than against each other (28,733). This is despite the fact that Sscroffa11.1 and Sscrofa10.2 represent the same pig genome. While some differences between Sscrofa10.2 and Sscrofa11.1 may be due to differences in which haplotype has been captured in the assembly, the reduction in low quality and low coverage regions and the dramatic decrease in differences versus USMARCv1.0 leads us to conclude that the majority are improvements in the assembly of Sscrofa11.1. The differences between the Sscrofa11.1 and USMARCv1.0 will represent a mix of true structural differences and assembly errors that will require further research to resolve.”

Close
