# Supplementary material for: An improved pig reference genome sequence to enable pig genetics and genomics research
Source: Gigascience. 2020 Jun 16;9(6):giaa051. doi: 10.1093/gigascience/giaa051 (PMC7448572; doi:10.1093/gigascience/giaa051)
Supplement: giaa051_Response_to_Reviewer_Comments_Revision_2 [file giaa051_response_to_reviewer_comments_revision_2.pdf]

# Author's Response To Reviewer Comments

Close

Dear Editors

I am pleased to submit a final revised version of the manuscript entitled “An improved pig reference genome sequence to enable pig genetics and genomics research”.

The questions and issues raised by the reviewers have been addressed as described previously.

The results from the Assemblytics comparisons of the 13 pig genome assemblies available in GigaDB (http://dx.doi.org/10.5524/100732).

The figures are integrated into the manuscript and supplementary materials. I have also uploaded copies of each figure image. These images are also available in the associated GigaDB dataset (http://dx.doi.org/10.5524/100732).

Yours sincerely

Alan L. Archibald

Close
