# Supplementary material for: An improved pig reference genome sequence to enable pig genetics and genomics research
Source: Gigascience. 2020 Jun 16;9(6):giaa051. doi: 10.1093/gigascience/giaa051 (PMC7448572; doi:10.1093/gigascience/giaa051)
Supplement: giaa051_Reviewer_1_Report_Original_Submission — Mingzhou Li -- 12/9/2019 Reviewed [file giaa051_reviewer_1_report_original_submission.pdf]

Reviewer Report

Title: An improved pig reference genome sequence to enable pig genetics and genomics research

Version: Original Submission **Date:** 12/9/2019

Reviewer name: Mingzhou Li

Reviewer Comments to Author:

The domestic pig is of enormous agricultural significance and valuable models for many human diseases. Nonetheless, the draft assembly of the reference pig genome (Sscrofa10.2) was incomplete (at least 8% of the sequence is estimated to be missing from the assembly) and limited its utility. The MS entitled "An improved pig reference genome sequence to enable pig genetics and genomics research" reported two annotated highly contiguous chromosome-level genome assemblies (i.e., Sscrofa11.1 and USMARCv1.0) and also presented annotation of a further 11 short read assemblies of representative pig breeds in Europe and Asia. Especially, the updated Sscrofa11.1 (Contig N50 = 48.23 Mb, scaffold N50 = 88.23 Mb,) is substantively superior than the former version of Sscrofa10.2 (Contig N50 = 69.50 Kb, scaffold N50 = 576.01 Kb). To the best of my knowledge, this high-quality assembly of the reference pig genome (Sscrofa11.1, released at Dec 2016) had been widely adapted by the pig genomics community. I appreciate authors' significant efforts for the pig genomics community, which provide an unprecedented view of the genetic make-up of this important agricultural and biomedical model species. The quality of the presentation is excellent, the structure of the presentation is clear and there are a very small number of typographical errors. Overall the discussions and conclusions appear sound and objective.
Specific comments:
1) Lines 50-51 "The domestic pig (Sus scrofa) is important both as a food source and as a biomedical model with high anatomical and immunological similarity to humans".
It is well documented that, compared with rodent, pig is closely comparable to human in size, anatomy, physiology, metabolism, pathology and pharmacology. Why only highlight "immunological similarity" here? As well as in Line 72: "including responses to infectious diseases"
2) Line 123 "MARC1423004 which was a Duroc/Landrace/Yorkshire crossbred barrow (i.e. castrated male pig)" . Is it means the terminal crossbreeding system with three pig breeds, i.e., Duroc Ã— (Landrace Ã— Yorkshire) (DLY). I think the author should provide the accurate description.
3) Lines 220-221 "After correcting the orientation of these inverted scaffolds, there is good agreement between the USMARCv1.0 assembly and the RH map (Fig. 1b)." I suggest the author should provide the exact statistic number to support the statement of "good agreement".
4ï¼‰Lines 286-287: "There were five genes that were present in the Iso-Seq data, but missing in the Sscrofa11.1 assembly.". I have not find the corresponding description of the method and the more detail results of the "identification of missing genes in the assembly" . I think the author should provide these essential information. Given the volume of information available, it is difficult to assess the methodology.
5) Lines 548-549: "haplotype resolved assemblies of a Meishan and White Composite F1 crossbred pig

currently being sequenced." Same as my comment 2), the author should accurately provide description of the sample.

Methods

Are the methods appropriate to the aims of the study, are they well described, and are necessary controls included? Choose an item.

Conclusions

Are the conclusions adequately supported by the data shown? Choose an item.

Reporting Standards

Does the manuscript adhere to the journal's guidelines on minimum standards of reporting? Choose an item.

Choose an item.

Statistics

Are you able to assess all statistics in the manuscript, including the appropriateness of statistical tests used? Choose an item.

Quality of Written English

Please indicate the quality of language in the manuscript: Choose an item.

Declaration of Competing Interests

Please complete a declaration of competing interests, considering the following questions:

- Have you in the past five years received reimbursements, fees, funding, or salary from an organisation that may in any way gain or lose financially from the publication of this manuscript, either now or in the future?
- Do you hold any stocks or shares in an organisation that may in any way gain or lose financially from the publication of this manuscript, either now or in the future?
- Do you hold or are you currently applying for any patents relating to the content of the manuscript?
- Have you received reimbursements, fees, funding, or salary from an organization that holds or has applied for patents relating to the content of the manuscript?
- Do you have any other financial competing interests?
- Do you have any non-financial competing interests in relation to this paper?

If you can answer no to all of the above, write 'I declare that I have no competing interests' below. If your reply is yes to any, please give details below.

I declare that I have no competing interests

I agree to the open peer review policy of the journal. I understand that my name will be included on my report to the authors and, if the manuscript is accepted for publication, my named report including any attachments I upload will be posted on the website along with the authors' responses. I agree for my report to be made available under an Open Access Creative Commons CC-BY license (http://creativecommons.org/licenses/by/4.0/). I understand that any comments which I do not wish to be included in my named report can be included as confidential comments to the editors, which will not be published.

Choose an item.

To further support our reviewers, we have joined with Publons, where you can gain additional credit to further highlight your hard work (see: https://publons.com/journal/530/gigascience). On publication of this paper, your review will be automatically added to Publons, you can then choose whether or not to claim your Publons credit. I understand this statement.

Yes Choose an item.
