# Supplementary material for: An improved pig reference genome sequence to enable pig genetics and genomics research
Source: Gigascience. 2020 Jun 16;9(6):giaa051. doi: 10.1093/gigascience/giaa051 (PMC7448572; doi:10.1093/gigascience/giaa051)
Supplement: giaa051_Reviewer_2_Report_Original_Submission — Shanlin Liu -- 12/15/2019 Reviewed [file giaa051_reviewer_2_report_original_submission.pdf]

Reviewer Report

Title: An improved pig reference genome sequence to enable pig genetics and genomics research

Version: Original Submission **Date:** 12/15/2019

Reviewer name: Shanlin Liu

Reviewer Comments to Author:

The authors present us with two high-quality genome assemblies for the pig. In addition to the regular assembly procedure to obtain the two assemblies, they have made great efforts to check the accuracies of both using lots of other datasets, including FISH, radiation hybrid map, BAC clones. I only have several minor concerns as follows:
The authors annotated both the genomes using full-length transcriptome data from a single individual. I wonder whether you have any specific filtering step to avoid incorrect annotations, as the differential expression (both expression level and alternative splicing) may contribute to their phenotypic variances.
Line 180 - 190, the authors may want to explain more on the definition of low quality and low coverage regions, e.g. What're your criteria? Besides, please provide statistics of GC content for those remaining LQLC regions to show your points of view better.
For the assembly of USMARC, the authors mentioned that " The resulting assemblies were compared and the Celera Assembler result was selected based on better agreement with a Dovetail ChicagoÂ® library," it is better to explain more on your definition for the "better agreement".
Line 235 - 245, identify heterozygous structure variances using long reads can check whether the incongruencies between the v11.1 and v10.2 derived from innate differences between two haploids.

Methods

Are the methods appropriate to the aims of the study, are they well described, and are necessary controls included? Choose an item.

Conclusions

Are the conclusions adequately supported by the data shown? Choose an item.

Reporting Standards

Does the manuscript adhere to the journal's guidelines on minimum standards of reporting? Choose an item.

Choose an item.

Statistics

Are you able to assess all statistics in the manuscript, including the appropriateness of statistical tests used? Choose an item.

Quality of Written English

Please indicate the quality of language in the manuscript: Choose an item.

Declaration of Competing Interests

Please complete a declaration of competing interests, considering the following questions:

- Have you in the past five years received reimbursements, fees, funding, or salary from an organisation that may in any way gain or lose financially from the publication of this manuscript, either now or in the future?
- Do you hold any stocks or shares in an organisation that may in any way gain or lose financially from the publication of this manuscript, either now or in the future?
- Do you hold or are you currently applying for any patents relating to the content of the manuscript?
- Have you received reimbursements, fees, funding, or salary from an organization that holds or has applied for patents relating to the content of the manuscript?
- Do you have any other financial competing interests?
- Do you have any non-financial competing interests in relation to this paper?

If you can answer no to all of the above, write 'I declare that I have no competing interests' below. If your reply is yes to any, please give details below.

I declare that I have no competing interests

I agree to the open peer review policy of the journal. I understand that my name will be included on my report to the authors and, if the manuscript is accepted for publication, my named report including any attachments I upload will be posted on the website along with the authors' responses. I agree for my report to be made available under an Open Access Creative Commons CC-BY license (http://creativecommons.org/licenses/by/4.0/). I understand that any comments which I do not wish to be included in my named report can be included as confidential comments to the editors, which will not be published.

Choose an item.

To further support our reviewers, we have joined with Publons, where you can gain additional credit to further highlight your hard work (see: https://publons.com/journal/530/gigascience). On publication of this paper, your review will be automatically added to Publons, you can then choose whether or not to claim your Publons credit. I understand this statement.

Yes Choose an item.
